# Targeted Proteomics Reveals Quantitative Differences in Low-Abundance Glycosyltransferases of Patients with Congenital Disorders of Glycosylation

**DOI:** 10.3390/ijms25021191

**Published:** 2024-01-18

**Authors:** Roman Sakson, Lars Beedgen, Patrick Bernhard, K. Merve Alp, Nicole Lübbehusen, Ralph Röth, Beate Niesler, Marcin Luzarowski, Olga Shevchuk, Matthias P. Mayer, Christian Thiel, Thomas Ruppert

**Affiliations:** 1 Zentrum für Molekulare Biologie der Universität Heidelberg (ZMBH), DKFZ-ZMBH Alliance, 69120 Heidelberg, Germany; 2 Heidelberg Biosciences International Graduate School (HBIGS), Heidelberg University, 69120 Heidelberg, Germany; 3 Center for Child and Adolescent Medicine, Department Pediatrics I, Heidelberg University, 69120 Heidelberg, Germany; 4 Institute for Surgical Pathology, Medical Center—University of Freiburg, Faculty of Medicine, University of Freiburg, 79106 Freiburg, Germany; 5 Max Delbrück Center for Molecular Medicine in the Helmholtz Association (MDC), 13125 Berlin, Germany; 6 nCounter Core Facility, Institute of Human Genetics, University Hospital Heidelberg, 69120 Heidelberg, Germany; 7 Interdisciplinary Center for Neurosciences, Heidelberg University, 69120 Heidelberg, Germany; 8 Department of Immunodynamics, Institute of Experimental Immunology and Imaging, University Hospital Essen, 45147 Essen, Germany

**Keywords:** glycosylation, proteomics, endoplasmic reticulum, congenital disorders of glycosylation, MRM, nCounter, Skyline

## Abstract

Protein glycosylation is an essential post-translational modification in all domains of life. Its impairment in humans can result in severe diseases named congenital disorders of glycosylation (CDGs). Most of the glycosyltransferases (GTs) responsible for proper glycosylation are polytopic membrane proteins that represent challenging targets in proteomics. We established a multiple reaction monitoring (MRM) assay to comprehensively quantify GTs involved in the processes of *N*-glycosylation and *O*- and *C*-mannosylation in the endoplasmic reticulum. High robustness was achieved by using an enriched membrane protein fraction of isotopically labeled HEK 293T cells as an internal protein standard. The analysis of primary skin fibroblasts from eight CDG type I patients with impaired *ALG1*, *ALG2*, and *ALG11* genes, respectively, revealed a substantial reduction in the corresponding protein levels. The abundance of the other GTs, however, remained unchanged at the transcript and protein levels, indicating that there is no fail-safe mechanism for the early steps of glycosylation in the endoplasmic reticulum. The established MRM assay was shared with the scientific community via the commonly used open source Skyline software environment, including Skyline Batch for automated data analysis. We demonstrate that another research group could easily reproduce all analysis steps, even while using different LC-MS hardware.

## 1. Introduction

**Figure 1:**
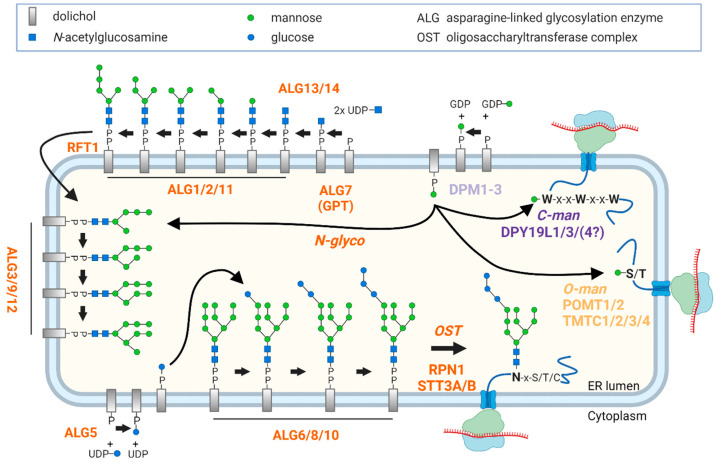
*N*-glycosylation, *O*-mannosylation, and *C*-mannosylation in the ER: For *N*-glycosylation, the *N*-glycan precursor is generated through the sequential addition of 14 carbohydrate moieties to dolichol phosphate by the ALG GTs and it is subsequently transferred en bloc to the *N*-glycosylation site of a nascent protein by the OST complex. Functional units, such as the three cytoplasmic mannosyltransferases ALG1, ALG2, and ALG11, are indicated as ALG1/2/11. Donor substrates at the cytoplasmic side of the ER are nucleotide-activated glycans, whereas lipid-linked glycans, Dol-P-Man and Dol-P-Glc, are used in the lumen of the ER. Created with BioRender.com.

Protein glycosylation is an essential modification conserved across all domains of life. Defects within the process of protein glycosylation in humans lead to the disease group congenital disorders of glycosylation (CDGs) [1]. For *N*-glycosylation, the most common form of glycosylation in mammals [2], a series of glycosyltransferases (GTs) catalyze the biosynthesis of a dolichol-linked oligosaccharide, which is then transferred en bloc onto acceptor proteins. The biosynthesis of this precursor starts with the addition of two *N*-acetylglucosamine residues to dolichol phosphate at the cytoplasmic side of the endoplasmic reticulum (ER) (Figure 1). Then, five mannose residues are sequentially attached, catalyzed by the mannosyltransferases ALG1, ALG2, and ALG11. Studies on yeast have demonstrated that these three enzymes form heteromeric complexes [3,4]. After flipping to the luminal side of the ER by the help of RFT1, the functional units consisting of ALG3, ALG9, ALG12 and ALG6, ALG8, ALG10 are necessary to complete the glycan structure of the precursor using dolichol monophosphate-activated mannose (Dol-P-Man) as a substrate [5] (Figure 1). This glycan structure is subsequently transferred en bloc from the precursor to the *N*-glycosylation site of the nascent protein by the oligosaccharyltransferase (OST) complex [6]. *N*-glycosylation shares the same substrate, Dol-P-Man, with two other types of glycosylation, which start in the ER, namely protein *O*-mannosylation and protein *C*-mannosylation. These two types are mediated by protein *O*-mannosyltransferases (POMT1/2, TMTC1/2/3/4) and *C*-mannosyltransferases (DPY19L1/3/4) (Figure 1) [7,8]. All these glycosylation pathways were mainly characterized by yeast genetics because most of the GTs involved in ER-based *N*-glycosylation and *O*- and *C*-mannosylation are polytopic membrane proteins, and are therefore challenging targets for quantitative analysis at the protein level [9].

Multiple reaction monitoring (MRM) of peptides is routinely used as a targeted proteomics strategy for the high-confidence identification and quantification of proteins in complex biological samples [10]. Skyline is an established open source software package widely used in this field, because it facilitates method development as well as peptide quantification [11]. Importantly, Skyline is compatible with mass spectrometer software from many different manufacturers. Skyline uses indexed retention times (iRTs) that are calculated from measured retention times relative to a set of predefined standard iRT peptides [12]. This allows Skyline to predict (or schedule) retention times and MRM transition windows for all peptides based on the retention times of the standard iRT peptides detected in a first unscheduled analysis on a new LC-MS system. The exported transition list is then used to measure the samples. This makes a scheduled MRM assay independent of the LC-MS system used. In addition, data processing in Skyline can also be automated. With the graphical user interface Skyline Batch (SB GUI), built on top of the Skyline command-line interface, users can configure an automated Skyline data processing workflow [13]. Such a configuration file contains all processing and analysis steps of an experiment. Therefore, it can be easily used to reprocess data with alternative parameters or to share all experimental details with the scientific community.

We describe the development of a comprehensive MRM assay to quantitatively study ER-resident GTs in human skin fibroblasts using a spike-in SILAC approach [14]. Our focus was on a panel of eight CDG type I patients carrying mutations in *ALG1*, *ALG2*, and *ALG11* genes, respectively, as studies on yeast showed that mutations in any of these genes lead to inviability or severe growth defects [15,16,17]. An analysis of human primary skin fibroblasts showed that, in most cases, mutations led to the almost complete loss of the corresponding protein. However, the loss of GTs did not trigger any regulatory event like increasing their transcript levels or changing the transcript or protein abundances of any other ALG enzyme from this essential biosynthesis pathway. An important technical aspect is that the described MRM assay is easily accessible to the scientific community, as in addition to peptide-specific device settings, such as transitions and predicted retention times, a script for automatic data processing has now been integrated into the Skyline software ecosystem using SB GUI.

## 2. Results

### 2.1. MRM Assay for the Quantitative Analysis of ER-Based GTs

**Figure 2:**
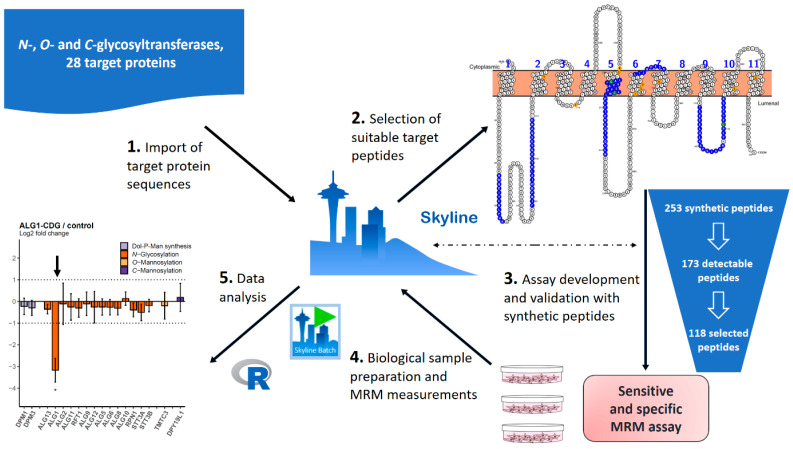
Schematic workflow for establishment and usage of the MRM assay in this study. Selected protein sequences were imported into Skyline and suitable peptides were selected, also considering known mutations, which is facilitated by the Skyline plugin Protter. Using synthetic peptides in a complex background, appropriate transitions were selected and iRTs calculated. All information is stored within a single Skyline document. Data were measured in a 1 h gradient and analyzed in an automated way using a newly established R-script via the SB GUI.

The major goal of this study was to develop a quantitative assay for the components of the glycosylation machinery in the human ER (Figure 1). We set up an MRM assay using the framework of the Skyline software environment. The overall workflow is shown in Figure 2. Most of the investigated GTs are ER-resident proteins with several transmembrane domains that often represent challenging targets for proteolytic digestion due to their physico-chemical properties [18]. Therefore, three to thirteen candidate peptides (median = 9) per protein were selected after examination for reported natural variants and modifications using the Skyline plugin Protter (version 1.0.1) [19]. Instrument parameters were optimized using commercially available heavy-labeled synthetic peptides for all selected peptide candidates. Out of 253 ordered synthetic peptides, 211 were successfully validated via the MRM-initiated detection and sequencing (MIDAS) approach (see Supplementary Materials for details) [20]. Those peptides were further tested in a representative background matrix consisting of a human cell lysate. This procedure led to a ready-to-use MRM assay that contained 173 peptides for 28 target proteins, as well as for proteins for quality control (PMM2) and data normalization (SEC63) (Figure 2, Table 1 and Table S1). We determined the indexed retention times (iRTs) for all peptides present in the MRM assay, which greatly facilitated the transfer of the method to a different laboratory, as described below in more detail [21]. All information about transitions and iRTs is included in a single Skyline document as a chromatogram library [22]. The result is a robust and adaptable assay ready for application in diverse laboratory settings.

An isotopically labeled internal protein standard allows for the compensation of experimental variability during sample processing [23]. Because the labeling of primary fibroblasts proved to be less efficient, we used the microsomal ER membrane fraction derived from an HEK 293T cell culture [24]. Such a membrane fraction of heavy-labeled HEK 293T cells contained most of the target proteins, with an approx. 8-fold enrichment compared to the whole-cell lysate (Figures S1 and S2 in Supplementary Materials). The addition of this internal standard only slightly increased the complexity of the samples to be analyzed, resulting in more interference-free transitions and better signal-to-noise ratios. Based on the signals from the internal standard, we selected 118 out of the 173 peptides mentioned above (on average, 4–5 peptides per protein) as well as corresponding transitions. This allowed us to monitor all target proteins within one scheduled LC-MRM measurement in a 1 h gradient, underlining the efficiency of our analytical approach.

Skyline not only allows the export of a list containing transitions, iRTs, and optimized collision energies to control instruments from different manufacturers for scheduled MRM data acquisition, but it also enables the import of acquired data back into the same Skyline document. This facilitates peak picking and quantification according to the specified transition list. We developed an R-script for downstream quantitative and statistical analysis for pairwise group comparisons (see Table S2 for details). With the SB GUI, it is now possible to streamline the whole data analysis process (see Supplementary Materials for details).

### 2.2. Relative Abundances of GTs in HEK 293T, HeLa, and Primary Skin Fibroblast Cell Lines

**Figure 3:**
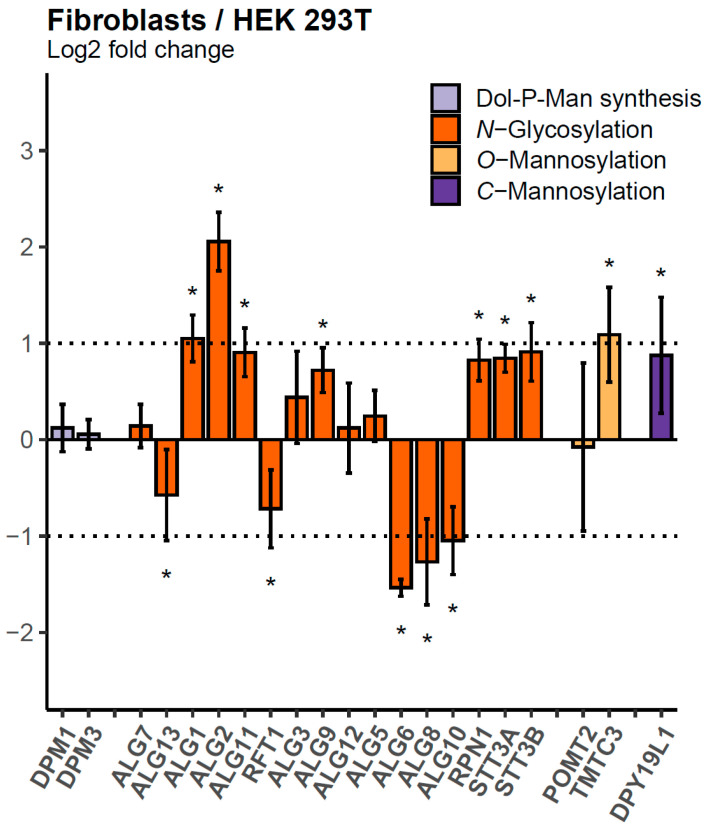
Relative quantification of 21 target proteins in human fibroblasts (n = 3) compared with HEK 293T cells (n = 3). Ratios between whole-cell lysates and the internal standard were obtained and used to calculate fold changes between fibroblasts and HEK 293T. Dotted lines indicate the range between + and − two-fold change (logarithmic scale) in relative protein abundance normalized to SEC63. Error bars show 95% confidence intervals and stars indicate adjusted *p*-values ≤ 0.05.

In a SILAC-based proteomics analysis, the sample and control are concurrently cultured in the “light” and “heavy” SILAC mediums before being combined and analyzed. To facilitate comparison between several samples, a heavy-labeled control stock can be produced. As a spike-in SILAC approach, an equal amount of labeled control is then added to each sample grown in a normal medium [14]. We tested whether an enriched membrane fraction of a different cell type, namely HEK 293T cells, can be used as an internal standard for the analysis of GTs in human primary fibroblasts, but also in other human cell lines. An aliquot of this membrane fraction was added to whole-cell lysates from HEK 293T and HeLa cell lines, as well as from three primary human skin fibroblast cell lines that were considered biological replicates. After tryptic digestion, 21 target proteins involved in *N*-glycosylation as well as *O*- and *C*-mannosylation and Dol-P-Man synthesis could be quantified (Figure 3). After normalization to SEC63, a subunit of the translocon complex, only minor differences in relative protein amounts (≤2-fold) were observed for most of the target proteins, which suggests that the HEK 293T-derived internal standard can be used to analyze human fibroblasts (Figure 3) and HeLa cells (Figure S3). There were, nevertheless, subtle yet significant differences observed between the HEK 293T and fibroblasts, and it is interesting to note that the members of functional units formed by ALG1, ALG2, ALG11 or ALG6, ALG8, ALG10 were collectively up- or downregulated, respectively. These findings underscore the potential utility of the HEK 293T-derived internal standard while highlighting subtle distinctions between cell types. The MRM assay was then used to analyze the abundance of GTs in primary skin fibroblasts of CDG type I patients.

### 2.3. Comparison of Protein and mRNA Abundance between CDG Patient Fibroblasts and Controls

Defects in the generation of the *N*-glycan precursor can result in rare, severe developmental disorders in humans called CDGs. In patient diagnostics, genetic analyses are increasingly used to elucidate the gene variants causing these diseases [25]. The MRM assay presented here allows us to quantify the impact of these gene variants on protein abundance. We analyzed fibroblasts originating from eight CDG type I patients carrying biallelic mutations in ALG1 (three patients), ALG2 (one patient), or ALG11 genes (four patients) (Table 2). A whole-cell lysate from primary fibroblasts from CDG patients and four healthy controls were used to determine the protein abundances of ER-localized GTs. In addition, transcript levels were determined using nCounter^®^ technology, Seattle, WA, USA.

**Figure 4:**
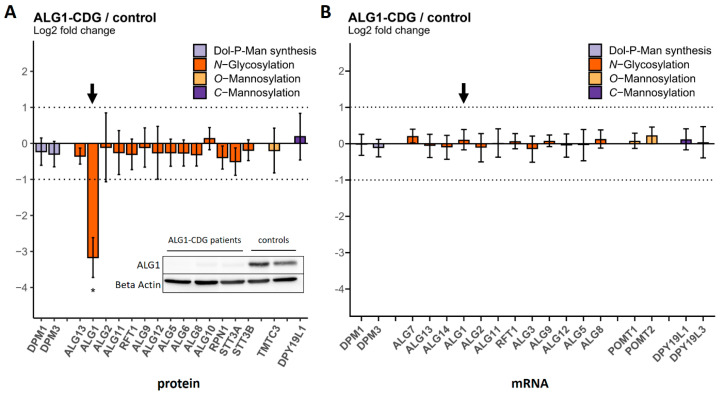
Protein and transcript levels of GTs in fibroblasts of ALG1−CDG patients (n = 3) compared to controls (n = 4). (**A**) Protein levels were determined via MRM. The ALG1 protein bar is highlighted with a black arrow. The inset shows the result of a Western blot. (**B**) mRNA transcript levels were determined using nCounter^®^ technology. The ALG1 mRNA transcript bar is highlighted with a black arrow. Dotted lines indicate the range between + and − two-fold change in relative abundance. Significant differences are labeled with an asterisk (adjusted *p*-value ≤ 0.05). Error bars show 95% confidence intervals.

The three ALG1-CDG patients available for this study carry the same homozygous mutation that replaces a serine with a leucine (Table 2). Whereas the transcript levels of ALG1 were not affected by this mutation, the abundance of the ALG1 protein was reduced by about 90% in the fibroblasts of all three patients compared to the controls, with a signal intensity close to the background (Figure 4). This was also confirmed through a Western blot analysis (Figure 4 and Figure S4A). This result indicates that the mutation severely affects protein stability.

**Figure 5:**
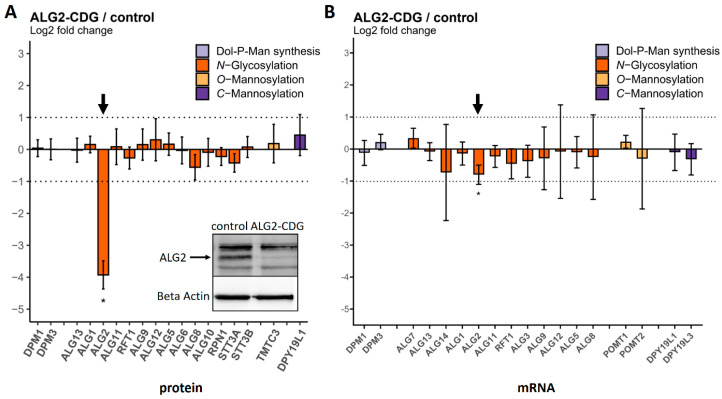
Protein and transcript levels of GTs in fibroblasts of the ALG2−CDG patient (n = 3 independent cell culture replicates) compared to controls (n = 4). (**A**) Protein levels were determined via MRM. The ALG2 protein bar is highlighted with a black arrow. The inset shows the result of a West-ern blot. (**B**) mRNA transcript levels were determined using nCounter^®^ technology. The ALG2 mRNA transcript bar is highlighted with a black arrow. Dotted lines indicate the range between + and − two-fold change in relative abundance. Significant differences are labeled with an asterisk (adjusted *p*-value ≤ 0.05). Error bars show 95% confidence intervals.

Similar results were obtained for the ALG2-CDG patient. The ALG2 transcript level was slightly decreased to approx. 60% of the control level, which is in agreement with previously published data [26]. At the protein level, however, the effect was much more pronounced, as the measured ALG2 abundance was at or below the detection limit. This observation aligns well with the findings from the Western blot analysis (Figure 5 and Figure S4B).

**Figure 6:**
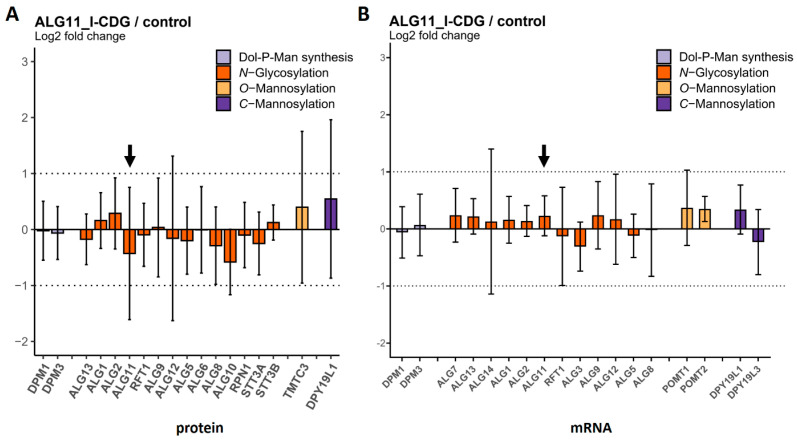
Protein and transcript levels of GTs in fibroblasts of the ALG11_I−CDG patient (n = 1) compared to controls (n = 4). (**A**) Protein levels were determined via MRM. The ALG11 protein bar is highlighted with a black arrow. (**B**) mRNA transcript levels were determined using nCounter^®^ technology. The ALG11 mRNA transcript bar is highlighted with a black arrow. Dotted lines indicate the range between + and − two-fold change in relative abundance. Error bars show 95% confidence intervals. No significant differences were observed.

**Figure 7:**
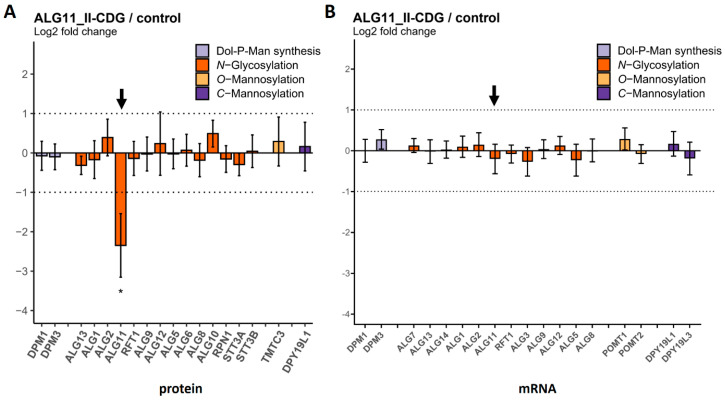
Protein and transcript levels of GTs in fibroblasts of ALG11_II−CDG patients (n = 3) com-pared to controls (n = 4). (**A**) Protein levels were determined via MRM. The ALG11 protein bar is highlighted with a black arrow. (**B**) mRNA transcript levels were determined using nCounter^®^ technology. The ALG11 mRNA transcript bar is highlighted with a black arrow. Dotted lines indicate the range between + and − two-fold change in relative abundance. Significant differences are labeled with an asterisk (adjusted *p*-value ≤ 0.05). Error bars show 95% confidence intervals.

We also analyzed fibroblasts from four ALG11-CDG patients carrying homozygous or heterozygous mutations (Table 2). In contrast to the results for ALG1-CDG and ALG2-CDG, the abundance of the ALG11 protein remained unchanged in one patient (referred to here as ALG11_I-CDG) (Figure 6). As the patient displayed a CDG phenotype [27], it is likely that the enzymatic function of the ALG11 protein was affected by the corresponding mutations. For the three other patients, the ALG11 protein was reduced to about 20% of the control levels (referred to as ALG11_II-CDG) (Figure 7). The mRNA levels remained unaffected in all four patients.

### 2.4. MRM Assay Results Were Easily Reproduced in a Cross-Laboratory Test

**Figure 8:**
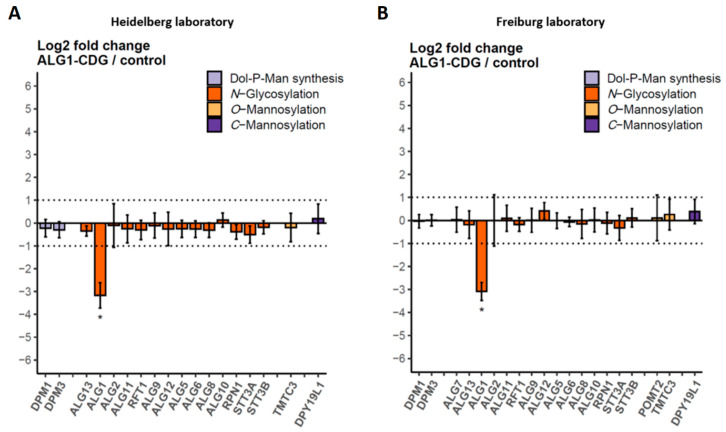
Reproducebility of the scheduled MRM assay between different laboratories. Cell pellets from ALG1-CDG samples (n = 3) and controls (n = 3), a sufficient amount of the internal protein standard, and the Skyline document with the MRM assay were used in a laboratory at the Universi-ty of Freiburg, Germany, to quantify the target proteins. Data acquired and processed in Heidelberg (**A**) and Freiburg (**B**) are shown. Dotted lines indicate the range between + and − two-fold change in relative protein abundance. Error bars show 95% confidence intervals for the calculated fold change and stars indicate adjusted *p*-values ≤ 0.05.

**Figure 9:**
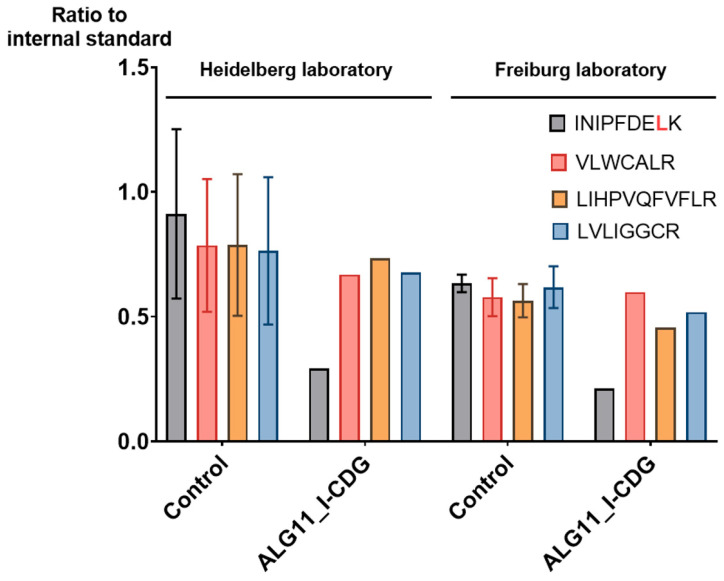
Evidence at the protein level for a single-nucleotide variant in the ALG11_I-CDG patient. ALG11 protein is reproducibly quantified by 4 peptides in Heidelberg and Freiburg. The 374-INIPFDELK-382 peptide harbors the single amino acid variant L381S. Only this peptide shows a decrease in signal intensity in the patient compared to the other three peptides and compared to healthy controls. The presence of the mutated peptide 374-INIPFDESK-382 in the patient sample was demonstrated in a separate MRM experiment (Figure S5).

Especially for clinical applications, an assay must be robust and easily shareable. The robustness of this scheduled MRM assay is strongly dependent on the labeled internal protein standard and the usage of iRTs based on 10 standard peptides. Data processing is also automated using the R-script, which is integrated within the SB GUI. To validate the usability and comparability of this MRM assay for different research groups, we provided cell pellets, a sufficient amount of the internal standard, and the Skyline document with the MRM assay to the group of Oliver Schilling at the University of Freiburg. Within one week, samples were processed and analyzed yielding results closely resembling those obtained in Heidelberg, although an LC-MS system from a different manufacturer was used (Figure 8). In addition, evidence of a single-nucleotide variant on one allele of the ALG11_I-CDG was found at the protein level in both laboratories. The single-nucleotide variant leads to an amino acid exchange L381S in the peptide 374-INIPFDELK-382. The wild-type (wt) peptide, monitored during the MRM assay, was reduced to approx. 35% compared to the controls, and may represent the translation of transcripts from the unaffected allele (Figure 9). In addition, the peptide 374-INIPFDESK-382 from the affected allele was detected in the respective ALG11 patient sample using a heavy-labeled synthetic peptide (Figure S5) as the standard.

## 3. Discussion

Protein glycosylation is an essential post-translational modification, and its impairment leads to severe diseases in humans, known as CDGs. Quantitative analyses of most GTs exhibit challenges due to their nature as low-abundance, polytopic membrane proteins [9,28]. To address this, we present a pipeline for the robust relative quantification of the major ER-resident GTs. This approach is facilitated by the Skyline software suite that controls not only data acquisition, but also subsequent data processing via an R-script integrated within the SB GUI [13]. Skyline is widely used by the scientific community. It conveniently collects all key parameters of the MRM assay within one document, encompassing information on proteins, selected peptides, transitions, optimized collision energies, and laboratory-independent iRTs, as well as chromatogram libraries. Eventually, all related data are uploaded to the respective Panorama Public repository project [22]. However, data processing is also an essential part of such a targeted analysis. Utilizing a custom report for quantitative data extraction from Skyline and an in-house written R-script for downstream data analysis, we further automated data processing through the use of the recently published SB GUI. This tool expedites data analysis without requiring expertise in the R programming language. SB GUI not only allows us to share the complete method with all settings for target proteins between laboratories and instrument platforms, but also enables data processing in a reproducible way. It is important to note that complementing the automated data import and analysis through SB GUI through the control of peak picking and integration with a human expert is still possible at any step.

To enable high-throughput analyses and to achieve high reproducibility, whole-cell lysates of human fibroblasts without further enrichment were used to quantify GTs. To reduce the variability in quantification even further, an isotopically labeled protein standard was added to each sample very early during sample processing [29]. However, the production of SILAC-labeled human fibroblasts turned out to be challenging. Furthermore, the use of a whole-cell lysate as an internal standard has the disadvantage that it increases the complexity of the sample to be analyzed by a factor of two, raising the risk of transition interferences by unrelated fragments. Therefore, we employed the HEK 293T cell culture model and an established method for the preparation of microsomal membranes. We showed that the relative amounts of most ER-resident GTs from the HEK 293T cells and from primary human fibroblasts are quite similar, which was a prerequisite to use the heavy-labeled enriched membrane fraction from HEK 293T cells. It is interesting to note that the relative amounts between the different GTs are also quite similar for HeLa cells. It is tempting to speculate that the GTs, as enzymes of this essential pathway, are conserved in terms of their absolute amounts across different cell lines.

The combination of an internal protein standard, iRTs, and software integration allowed us to demonstrate, in collaboration with a research group at Freiburg University, that the protein samples analyzed in both laboratories were highly comparable, even though LC-MS systems from different manufacturers were used. The overall time investment of the research group in Freiburg for analyzing the samples was less than a week from receiving the cell lysates to completing the analysis.

All ALGs except ALG3 involved in the synthesis of the *N*-glycan precursor were consistently quantified from a whole-cell lysate, but others like POMT1 and POMT2 were not even detected in many cases. They were below the limits of detection/quantification, which depend on instrumentation, the biological source, and enrichment strategies. For such different situations, these GTs are nevertheless included in the MRM assay.

In the clinical context, fibroblasts from eight CDG patients with severe phenotypes carrying mutations in both alleles of *ALG1*, *ALG2*, or *ALG11* were analyzed. Whilst having access to a higher number of CDG patients would have been desirable, most CDGs are very rare disorders [30]. In particular, there were only nine reported cases of ALG2-CDG worldwide until 2021, with the patient investigated in this study being the index patient [26,31,32,33]. The transcript levels of all GTs analyzed in this study were not affected in patient fibroblasts, except for a minor downregulation of the *ALG2* gene in the ALG2-CDG patient. This indicates that there is no mechanism that would sense severe problems during the early steps of *N*-glycosylation and try to compensate by regulating the abundance of responsible proteins at the transcript level. At the protein level, none of the analyzed GTs, except for those corresponding to the genetic defect, showed significant changes in abundance. Notably, the ALG1 and ALG2 protein abundance was reduced by at least 90% in the fibroblasts of CDG patients carrying mutations in the respective genes. Because the corresponding mRNA transcripts were present, the instability of the protein products would most likely cause rapid degradation.

In contrast, the ALG11 levels in the fibroblasts of the four ALG11-CDG patients were by far less strongly decreased, disclosing a different scenario. In one case, the ALG11 protein abundance was not affected at all, even though the case report described severe clinical characteristics [27]. This result indicates that, in this case, not the protein stability, but rather the enzymatic activity of ALG11, was affected. We could further confirm the reported genetic mutation in the ALG11_I-CDG patient at the peptide level using MRM [27]. The heterozygous mutation leads to a single amino acid exchange, L381S, within one of the four target peptides of ALG11. The wt, as well as the mutated peptide variant, could be detected. Whilst the relative amount of the other three peptides remained unchanged, the amount of the affected peptide from the same protein was significantly reduced. One possible explanation is that only 50% of the transcripts originate from the wt allele, whereas the other 50% are translated to the mutated peptide. Importantly, this effect was also precisely reproduced by the collaborating group in Freiburg and emphasized the reproducibility of the method across laboratories.

For three patients, ALG11 was reduced to approx. 20% of the control levels, which indicates that both allele products are less stable than the wt allele product. Since the mRNA levels were not changed, none of the mutations affect transcription. In contrast to the ALG1-CDG and ALG2-CDG patients, the relative protein amount of the affected GT was less drastically reduced in all ALG11-CDG patients.

Taken together, the developed MRM assay is easily shared with members of the scientific community. It allows us to study glycosylation processes in the ER by quantifying GTs from complex biological samples in a comprehensive way. The assay provides new insights into the effects of mutations causing CDG and represents a valuable tool for future research in this field, potentially advancing challenging diagnostic procedures for CDGs associated with new variants of uncertain significance.

## 4. Materials and Methods

### 4.1. Cell Culture and Protein Extraction

This study was performed in accordance with the Declaration of Helsinki and approved by the Ethics Committee of the Medical Faculty Heidelberg (S-904/2019). Written informed consent was obtained from the patients’ parents for the laboratory analysis of patient material. Fibroblasts and HEK 293T and HeLa cells were cultured in Dulbecco’s modified Eagle’s medium (DMEM) supplemented with 10% FCS, 100 U/mL of penicillin, and 100 µg/mL of streptomycin (see Supplementary Materials for details, including Table S3 regarding the used cell lines). For protein extract preparation, 80–90% confluent cell monolayers of a T75 flask were washed with ice-cold PBS, harvested, and stored at −80 °C.

For metabolic labeling, the HEK 293T cells were cultured in DMEM for SILAC (Thermo Fisher Scientific, Waltham, MA, USA) supplemented with 10% (*v*/*v*) FBS, 100 U/mL of penicillin, 100 µg/mL of streptomycin, 1% (*v*/*v*) GlutaMAX^®^ (Thermo Fisher Scientific), 0.55 mM of [^13^C_6_, ^15^N_4_]-arginine, and 0.82 mM of [^13^C_6_, ^15^N_2_]-lysine (Cambridge Isotopes, Tewksbury, MA, USA) for at least seven passages [34]. The cells were harvested and stored as described above. The preparation of the membrane protein fraction was performed as described (see Supplementary Materials for details) [24].

### 4.2. RNA Preparation and Nanostring/nCounter^®^ Analysis

Total RNA was extracted from 8 × 10^5^ cells with the RNeasy mini kit (Qiagen, Venlo, The Netherlands) in combination with the QIAshredder system (Qiagen) according to the manufacturer’s protocol. In short, the cells were lysed and then homogenized with the QIAshredder. Ethanol was added to the lysate to provide ideal binding conditions to the RNeasy silica membrane. After several washing steps, the RNA was eluted in 30 µL of nuclease-free H_2_O. The RNA quantity and quality were assessed using the NanoDrop 2000 (Thermo Fisher Scientific), the Agilent Bioanalyzer 2100 (Santa Clara, CA, USA) (TotalRNA Nanokit), and the Qubit™ Fluorometer (Qubit RNA HS Kit; Thermo Fisher Scientific). For each hybridization reaction, 50 ng of total RNA was used. The transcript abundance of 18 GT genes and control genes was determined using an nCounter^®^ SPRINT Profiler (NanoString Technologies, Seattle, WA, USA). For each patient and control fibroblast cell line, 3–4 replicates were analyzed (for raw data as well as for the nCounter^®^ probe design, see Table S4). Data were analyzed according to the manufacturer’s guidelines using the freely available nSolver Analysis Software 4.0. In short, the acquired data were normalized in two steps. First, a positive control normalization was performed by using a factor that was calculated based on the geometric mean of the positive controls that were added to every sample. Second, a housekeeping gene normalization was conducted by using the genes *C1orf43* and *SNRPD3*. From those normalized values, a ratio (fold change) from the patient-to-control sample means was calculated for every gene. All results were plotted with Prism 8.3.0.

### 4.3. Western Blot Analysis

For the Western blot, 10 μg of total protein derived from patient and control fibroblasts were used. The samples were mixed with 6x Laemmli buffer (375 mM Tris–HCl, pH 6.8, 6% SDS, 48% glycerol, 9% 2-mercaptoethanol, 0.03% bromophenol blue) and denatured at 95 °C for 5 min. The extracts were analyzed on a 12.5% SDS-PAGE and blotted onto a nitrocellulose membrane (GE Healthcare, Solingen, Germany) via semi-dry electrophoretic transfer. The membrane was blocked for 1 h at room temperature (RT) with 5% milk powder in PBST (0.1% Tween20 (SERVA; Heidelberg, Germany) in PBS). After blocking, the membrane was washed and incubated with the primary antibodies against ALG1 (Proteintech (Rosemont, IL, USA), 12872-1-AP, 1:1000 dilution) or ALG2 (Thermo Fisher Scientific, PA5-43263, 1:1000 dilution) overnight at 4 °C. After washing, the membrane was incubated with secondary antibody anti-rabbit IgG conjugated with horseradish peroxidase (Santa Cruz (Dallas, TX, USA); 1:10,000) for 45 min at RT. Protein signals were detected via light emission with a Pierce™ enhanced chemiluminescence reagent (ECL) Western blot analysis substrate (Thermo Fisher Scientific). After stripping the blots with 10% acetic acid for 15 min, the membrane was blocked again with 5% milk powder in PBST for 1 h. The membranes were incubated with primary antibodies against ß-actin (1:10,000, A5441, Sigma, St. Louis, MO, USA) for 1 h at RT. Then, the membranes were incubated with the secondary antibody anti-rabbit IgG conjugated with horseradish peroxidase for 45 min and detected with a Pierce ECL plus assay kit (Thermo Fisher Scientific). The Western blot data were normalized against the ß-actin signal and controls were set to 100%. All experiments were repeated a minimum of three times.

### 4.4. MRM Assay Development and Refinement

For each target protein, all unique tryptic peptides between 7 and 21 amino acids in length, not containing methionine, and without missed cleavages were considered (see Supplementary Materials for details). Heavy [^13^C_6_ ^15^N_2_]-lysine- and [^13^C_6_ ^15^N_4_]-arginine-labeled peptides were ordered from JPT Peptide Technologies (Berlin, Germany) and SynPeptide and contained carbamidomethyl-modified cysteine residues, if applicable. Peptides were analyzed using a Waters nanoACQUITY UPLC System (Waters, Milford, MA, USA), equipped with a trapping column (Waters, Symmetry C18; 2 cm length, 180 μm inner diameter, 5 μM C18 particle size, 100 Å pore size) and an analytical column (Waters, M-Class Peptide BEH C18; 25 cm length, 75 μm inner diameter, 1.7 μM C18 particle size, 130 Å pore size), which was coupled online to an ESI-QTrap 5500 via a NanoSpray III Source (Sciex). After trapping for 7 min at a flow rate of 10 µL/min with 99.4% of buffer A, separation was performed at 60 °C with a flow rate of 300 nL/min and a 37 min linear gradient of 3 to 37% buffer B (buffer A: 1% *v/v* ACN, 0.1% *v/v* FA; buffer B: 89.9% *v/v* ACN, 0.1% *v/v* FA). The MRM signals were validated using the MRM-initiated detection and sequencing (MIDAS) approach as described [20] with minor adaptations (see Supplementary Materials for details). A data analysis was performed in Skyline 20.1.0.155 [35]. iRTs were calculated using the iRT peptide standards [12]. Based on the MIDAS measurements, the 3–4 most intense transitions per precursor ion were selected. Collision energies were optimized for all transitions [36]. One pmol of each synthetic peptide was spiked into a mixture of three different human cell lysates (5 µg on column) to verify detectability in complex biological matrices. The final method is available on Panorama Public as a chromatogram library.

### 4.5. Sample Preparation and LC-MRM Measurements (Heidelberg)

Fibroblast cell pellets harvested from 80–90% confluent cell monolayers of a T75 flask were lysed in 100 µL RIPA buffer (Thermo Fisher Scientific) supplemented with 1% protease inhibitor mix (Roche) and 1 µL benzonase (250 units/µL, Merck). After incubation on ice for 30 min, cell lysate was passed 10-times through a 20 G needle and centrifuged for 30 min at 13,000 rpm at 4 °C. For HEK 293T and HeLa cells, the total amount of used benzonase was 750 units, and needle treatment was omitted. Protein concentration was determined with the DC protein assay (Bio-Rad). Cell lysates were shock-frozen in liquid nitrogen and stored at −80 °C until further analysis. Sample amounts corresponded to 31–40 µg of total protein, 20–28% (*w*/*w*) of which consisted of the internal standard, depending on the particular experiment. After spike-in of the internal standard, proteins were precipitated using a mixture of methanol and chloroform as described [37].

For in-solution digestion, precipitated protein pellets were resuspended in 20 µL of urea buffer (8 M urea, Carl Roth, 100 mM NaCl, Applichem, in 50 mM triethylammonium bicarbonate (TEAB), pH 8.5, Sigma-Aldrich). After reduction and alkylation by 10 mM Tris(2-carboxyethyl)phosphin (Carl Roth) and 40 mM 2-Chloroacetamide (Sigma-Aldrich) for 30 min at RT, 2.5 µg Lysyl Endopeptidase^®^ (Wako Chemicals) were added for 4 h at 37 °C. Urea was then diluted to 2 M using 50 mM TEAB buffer and 1 µg of trypsin (Thermo Fisher Scientific) was added. The digestion process was stopped after 16–17 h at 37 °C by the addition of trifluoroacetic acid (TFA, Sigma-Aldrich) to a final concentration of 0.4–0.8% (*v*/*v*). Samples were centrifuged for 10 min at 2500× *g* at RT and desalted using two C18 StageTips in parallel to avoid overloading the C18 material, with each StageTip containing 3 disks of Empore C18 material (3M) [38]. Eluted peptides were pooled, dried in a vacuum centrifuge and stored at −20 °C until LC-MRM analysis.

Peptides were resolubilized in 20% ACN/0.1% (*v*/*v*) TFA for 5 min at RT and then diluted 10-fold with 0.1% of TFA and further incubated for 1 h. Peptide solutions (0.5–1.0 µg µL^−1^) were immediately analyzed or stored at −20 °C. Between 8 to 9 μg of tryptic peptides were injected per LC-MRM analysis. LC-MS setup and gradient used were as described above for the assay development. All samples were measured in a randomized order.

### 4.6. Sample Preparation and LC-MRM Measurements (Freiburg)

In Freiburg, sample preparation was performed in the same way as in Heidelberg except that, for desalting, Hypersep C18 SpinTips (Thermo Fisher Scientific) were used. For LC-MRM, a nanoflow Easy-nLC II system (Proxeon Biosystems; Odense, Denmark) was used, equipped with a trapping column (Fused Silica Capillary; 3 cm length, JR-T-7360-100, VICI Jour, Schenkon, Switzerland) and with an analytical Self-Pack PicoFrit column (40 cm length, 75 μm inner diameter, New Objective (Littleton, MA, USA)), both columns in-house packed with C18 particles (Dr. Maisch (Ammerbuch-Entringen, Germany), ReproSil-Pur 120 C18-AQ; 3 μm C18 particle size, 120 Å pore size) [39]. The samples were trapped at 220 bar with 100% of buffer A and separated using a linear gradient of buffer B in buffer A from 8 to 56% of B in 60 min with a column temperature of 60 °C and a flow rate of 250 nL/min (buffer A: 0.1% *v/v* FA; buffer B: 50% *v/v* ACN, 0.1% *v/v* FA). The Easy-nLC II system was coupled online to an ESI-TSQ Vantage triple quadrupole mass spectrometer via a Nanospray Flex Ion source (both Thermo Scientific).

### 4.7. LC-MRM Data and Statistical Analysis

To establish the data analysis pipeline, the MRM data were processed manually using Skyline 20.1.0.155. The MRM data and Skyline documents have been deposited to the ProteomeXchange Consortium via the Panorama Public partner repository [22] with the dataset identifier PXD048072. After data import, the signals were reviewed using the Peak Areas Replicate Comparison visualization feature (Normalized to Total view). All peptides with reference dot-product (rdotp) correlations above 0.9 were selected. All transitions reported in Skyline as “Truncated”, “Fwhm Degenerate”, or not “Coeluting” were manually inspected. A transition was marked as not quantitative in the respective Skyline document via the document grid in cases when an obvious interference was detected upon manual inspection, thereby removing the transition signal from all samples for later quantification. All data were exported from Skyline using a customized document grid report template and imported in the csv-format into R version 4.2.2 (31 October 2022) “Innocent and Trusting” [40]. Data normalization, as well as the calculation of fold changes, adjusted *p*-values, and 95% confidence intervals, was performed with an in-house written R-script (Table S2). Briefly, all quantitative transitions were summed up across proteins, and log2 light/heavy ratios were calculated to minimize technical variance. In addition, all samples were normalized to their respective SEC63 protein level as a general ER marker membrane protein to account for biological variance [41]. Then, pairwise group comparisons were performed using linear regression models between protein ratios from different conditions and controls. The slope of the linear regression corresponded to the fold change between the respective condition and control. The respective standard error of linear regression was used to calculate the 95% confidence intervals. The computed *p*-values were adjusted using the Benjamini and Hochberg multiple test correction [42]. The R-code used for the pairwise group comparisons was inspired by the R package MSstats [43].

After preliminary testing, the described data analysis procedure was automated using SB GUI 21.2.1.389. The corresponding batch configuration file, as well as all necessary components, such as the Skyline template document containing the final transition list, the document grid custom report template, and the R-script used for data analysis, have been deposited to Panorama Public.

## Figures and Tables

**Table 1:** Overview of 30 proteins included in the MRM assay. Color code for *N-glycosylation***,** *O-mannosylation***,** *C-mannosylation,* and *Dol-P-Man synthesis* is used throughout all figures. Canonical sequences are meant, if there is no isoform specified. PMM2, as a cytosolic protein, was used as a quality control for membrane enrichment efficiency and excluded from further analysis.

Protein Class	Gene Name (UniProtKB Accession Number)
*N* -glycosylation	ALG7 (Q9H3H5); ALG13 (Q9NP73-2); ALG14 (Q96F25); ALG1 (Q9BT22); ALG2 (Q9H553); ALG11 (Q2TAA5); RFT1 (Q96AA3); ALG3 (Q92685); ALG9 (Q9H6U8); ALG12 (Q9BV10); ALG5 (Q9Y673); ALG6 (Q9Y672); ALG8 (Q9BVK2); ALG10 (Q5BKT4 or Q5I7T1); RPN1 (P04843); STT3A (P46977); STT3B (Q8TCJ2)
*O* -mannosylation	POMT1 (Q9Y6A1-2); POMT2 (Q9UKY4); TMTC1 (Q8IUR5); TMTC2 (Q8N394); TMTC3 (Q6ZXV5); TMTC4 (Q5T4D3)
*C* -mannosylation	DPY19L1 (A0A1B0GW05); DPY19L3 (Q6ZPD9); DPY19L4 (Q7Z388)
Dol-P-Man synthesis	DPM1 (O60762); DPM3 (Q9P2X0-2)
Nucleotide-activated sugars	PMM2 (O15305)
Housekeeping	SEC63 (Q9UGP8)

**Table 2:** Overview of known mutations in skin fibroblasts from CDG patients. All three ALG1-CDG patients carried the same homozygous mutation.

CDG	Number of Patients	Mutation
ALG1-CDG	3	homozygous c.C773T, p.S258L
ALG2-CDG	1	heterozygous c.1040delG p.G347VfsX26 c.G393T p.()
ALG11_I-CDG	1	heterozygous c.1142T > C/p.L381S and c.1192G > A/p.E398K
ALG11_II-CDG	3	homozygous c.A953C, p.Q318P; homozygous c.T257C, p.L86S heterozygous c.A836C p.Y279S and c.del623- 642;

## Data Availability

The MRM data have been deposited to the ProteomeXchange Consortium *via* the Panorama Public partner repository with the dataset identifier PXD048072.

## Supplementary Materials

The following supporting information can be downloaded at https://www.mdpi.com/article/10.3390/ijms25021191/s1. References [44,45,46] are cited in the Supplementary Materials.

## Abbreviations

CDGs: congenital disorders of glycosylation; DMEM: Dulbecco’s modified Eagle’s medium; Dol-P-Man: dolichol monophosphate-activated mannose; ECL: enhanced chemiluminescence reagent; ER: endoplasmic reticulum; GTs: glycosyltransferases; iRTs: indexed retention times; LC: liquid chromatography; MIDAS: MRM-initiated detection and sequencing; MRM: multiple reaction monitoring; MS: mass spectrometry; OST: oligosaccharyltransferase; RT: room temperature; SB GUI: graphical user interface, Skyline Batch; SILAC: stable isotope labeling by amino acids in cell culture; TEAB: triethylammonium bicarbonate; TFA: trifluoroacetic acid; WT: wild-type.
