# Supplementary material for: Targeted Proteomics Reveals Quantitative Differences in Low-Abundance Glycosyltransferases of Patients with Congenital Disorders of Glycosylation
Source: Int J Mol Sci. 2024 Jan 18;25(2):1191. doi: 10.3390/ijms25021191 (PMC10816918; doi:10.3390/ijms25021191)

Installation of necessary software:

To run the presented data analysis pipeline using the R-script as well as Skyline Batch (SB), you will need to install Skyline (version 20.2 or later), SB as well as R.

You can get a Skyline installation of your choice here:

https://skyline.ms/wiki/home/software/Skyline/page.view?name=install-64-disconnected_23_1

You can install SB from here:

https://skyline.ms/wiki/home/software/Skyline/page.view?name=skyline-batch

A current version of R can be obtained here:

https://cran.r-project.org/

Furthermore, we recommend to install RStudio after you have installed R, as it allows for an easy check whether all packages required by the provided R-script are also installed on your system (just open the R-script in RStudio before using it with Skyline Batch and RStudio will guide you through the installation process of necessary missing packages, if required):

https://posit.co/download/rstudio-desktop/

Getting started:

Please, consult the general SB documentation first:

https://skyline.gs.washington.edu/software/SkylineBatch/SkylineBatchDocumentation.pdf

All necessary files, such as the bcfg file, the "Skyline_Batch_Template" Skyline file and the skyr report for transition area export as well as the R-script have been deposited on Panorama Public (see main manuscript for accession details).

Below, screenshots of our SB configuration are provided to facilitate the setup of the pipeline:

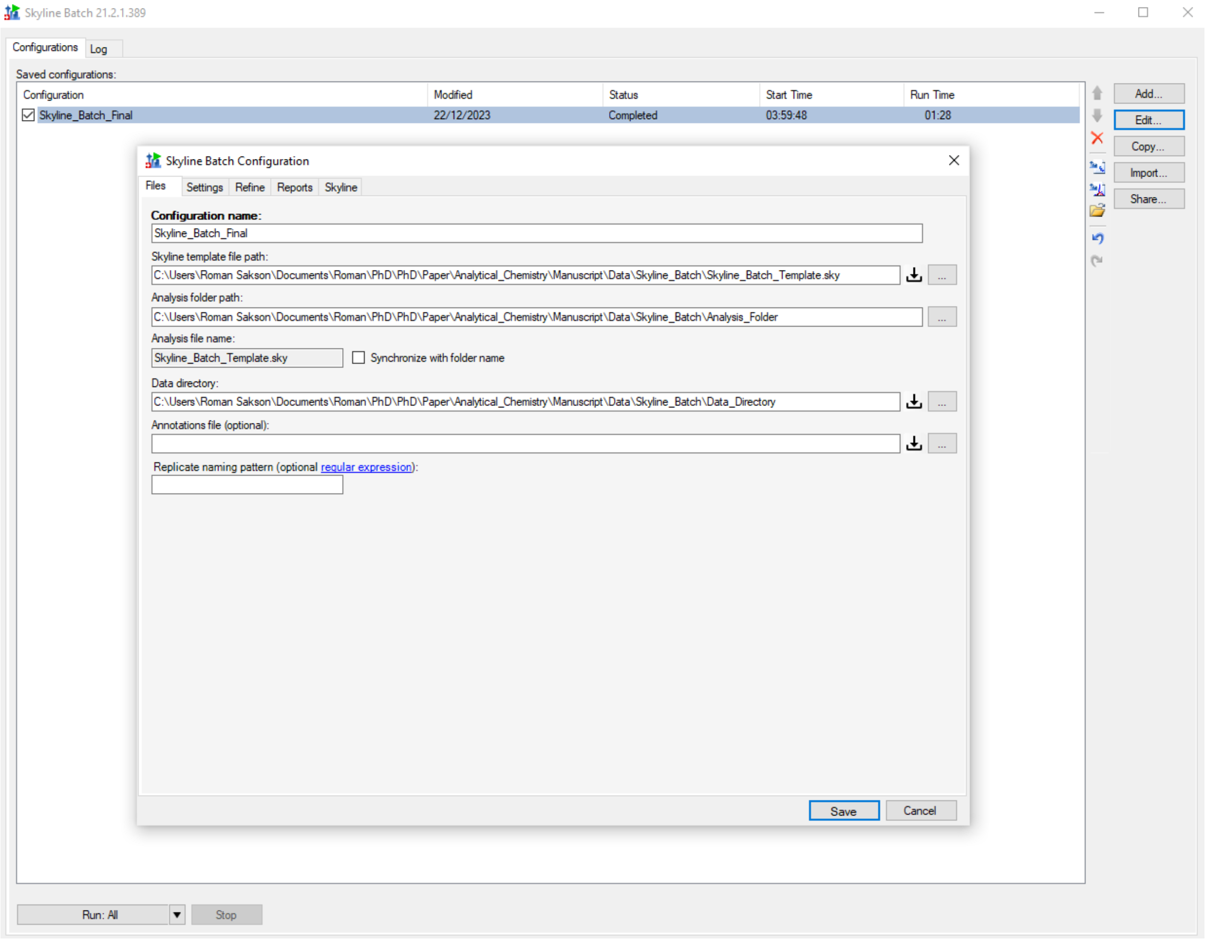

Settings and Refine options were not used:

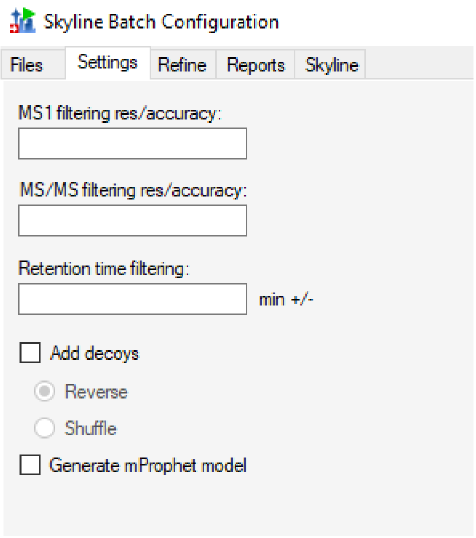

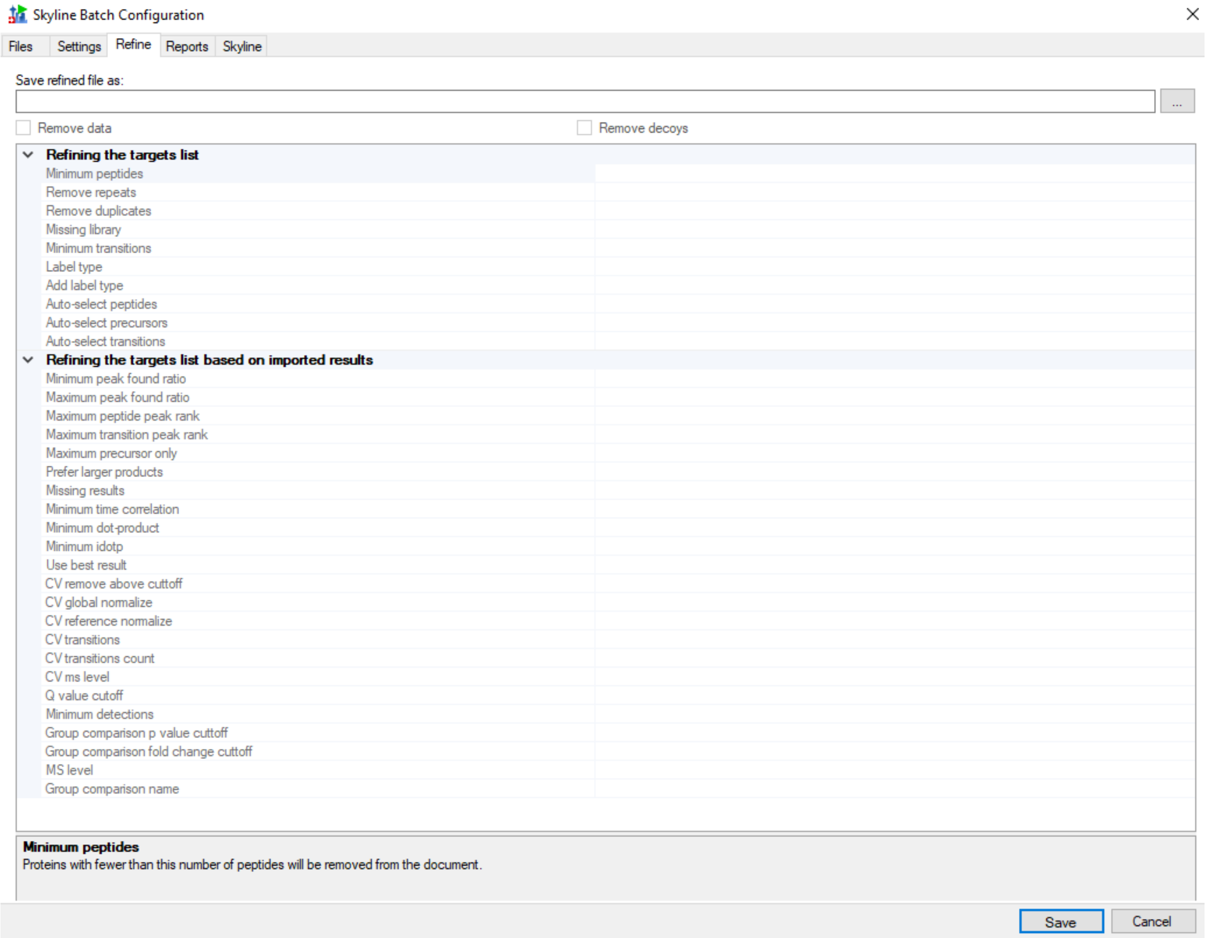
Skyline Batch Configuration
Files
Settings
Refine
Reports
Skyline
Save refined file as:
...
Remove data
Remove decoys
Refining the targets list
Minimum peptides
Remove repeats
Remove duplicates
Missing library
Minimum transitions
Label type
Add label type
Auto-select peptides
Auto-select precursors
Auto-select transitions
Refining the targets list based on imported results
Minimum peak found ratio
Maximum peak found ratio
Maximum peptide peak rank
Maximum transition peak rank
Maximum precursor only
Prefer larger products
Missing results
Minimum time correlation
Minimum dot-product
Minimum idotp
Use best result
CV remove above cuttoff
CV global normalize
CV reference normalize
CV transitions
CV transitions count
CV ms level
Q value cutoff
Minimum detections
Group comparison p value cuttoff
Group comparison fold change cuttoff
MS level
Group comparison name
Minimum peptides
Proteins with fewer than this number of peptides will be removed from the document.
Save
Cancel

Make sure to check the “culture-invariant report” box to avoid spaces in the column header names of the Skyline custom export report, otherwise the R-script will not run through:

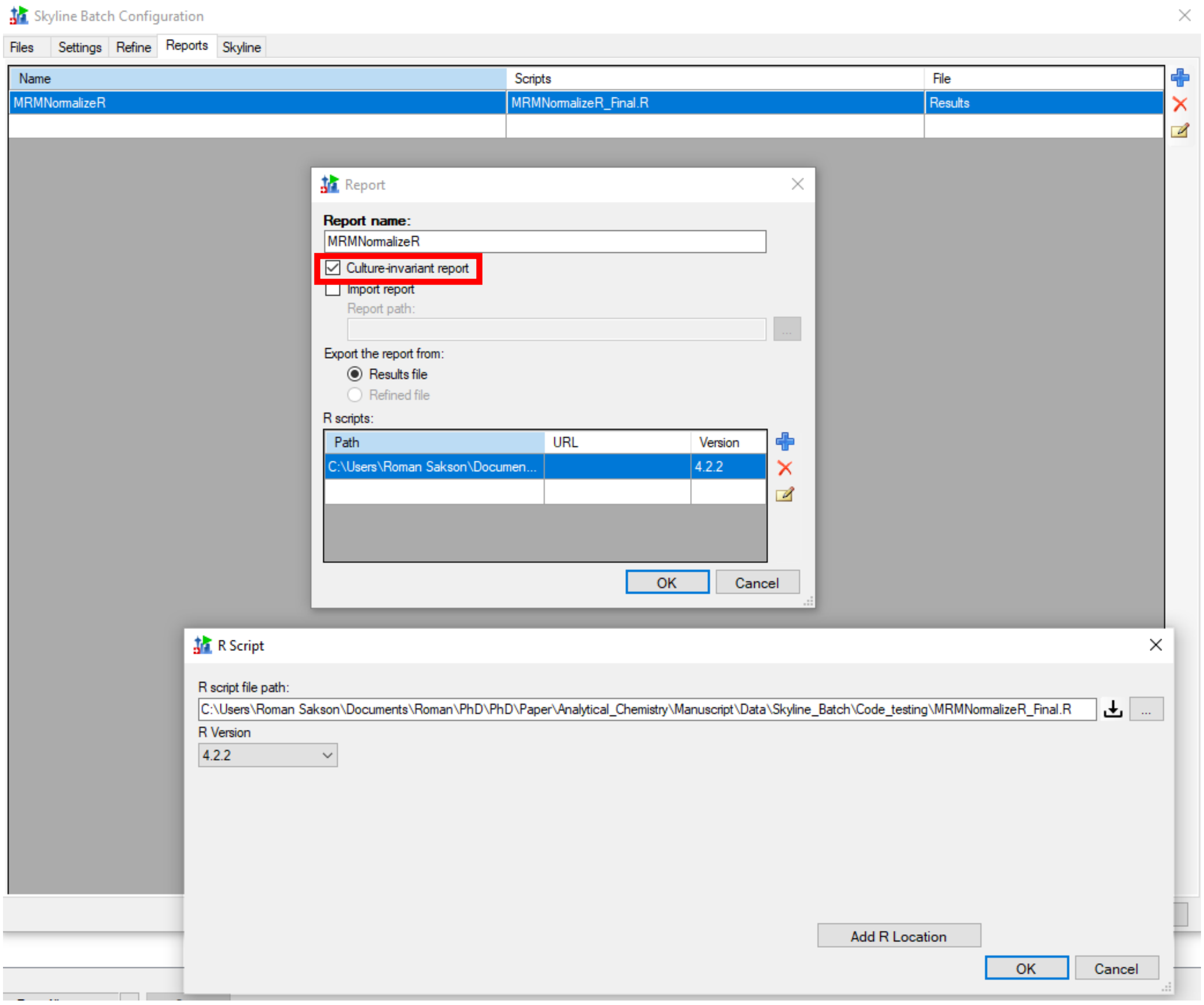

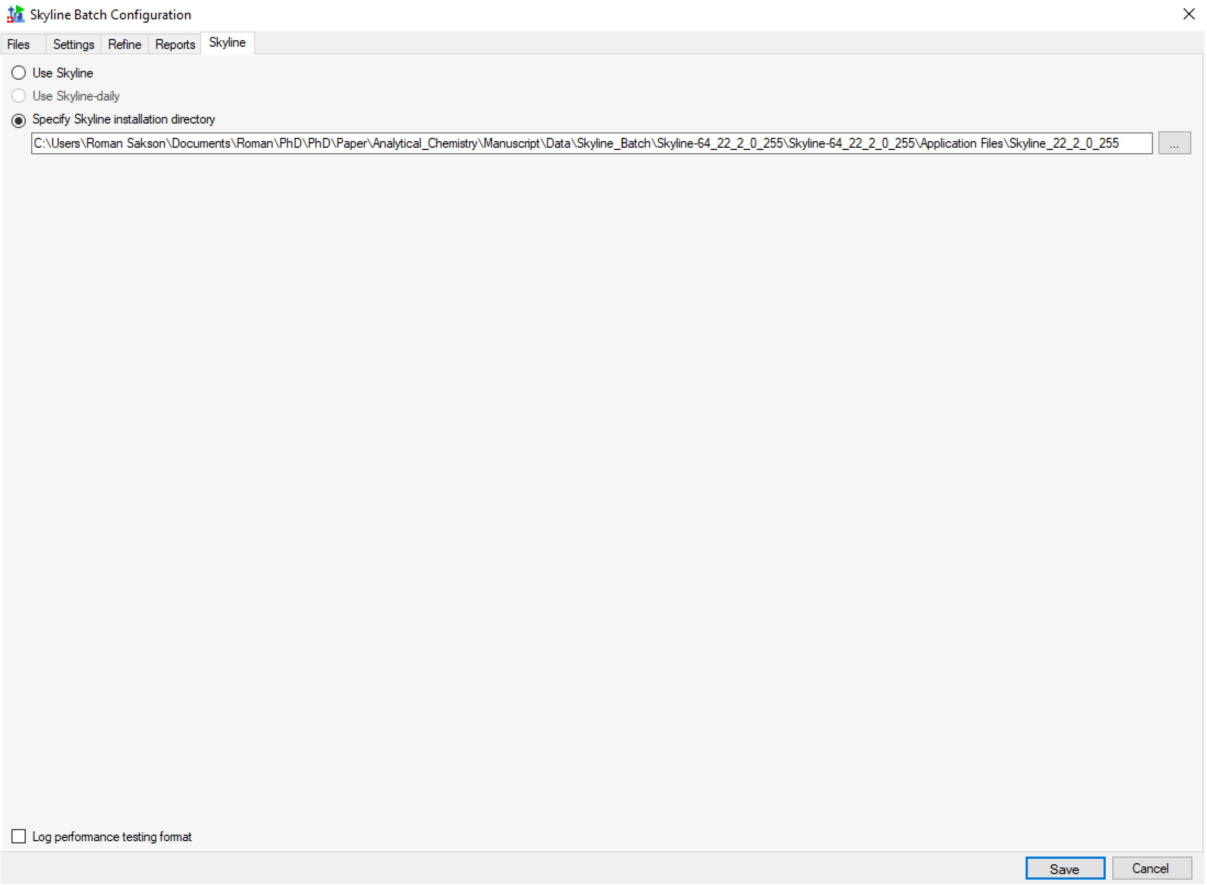

Here an example of a successful log record (note that there will be a pop-up fenster to set the working directory where your R-script is saved):

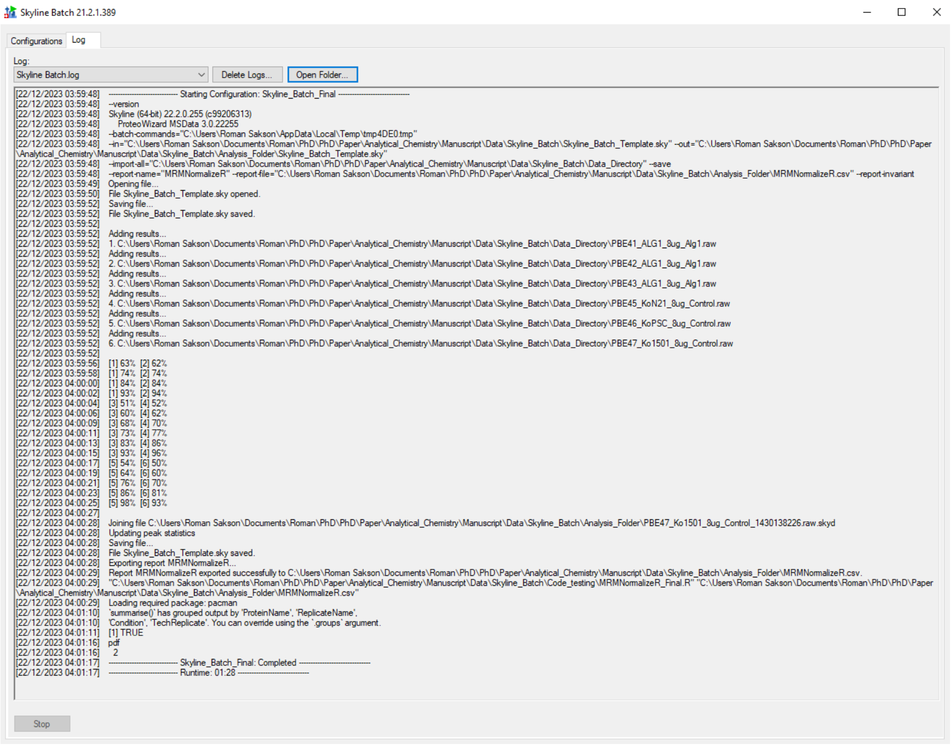

Supplement: Supplementary file 1 [file ijms-25-01191-s001.zip › Skyline Batch Guidelines.pdf]
