# Supplementary material for: Targeted Proteomics Reveals Quantitative Differences in Low-Abundance Glycosyltransferases of Patients with Congenital Disorders of Glycosylation
Source: Int J Mol Sci. 2024 Jan 18;25(2):1191. doi: 10.3390/ijms25021191 (PMC10816918; doi:10.3390/ijms25021191)
Supplement: Supplementary file 1 [file ijms-25-01191-s001.zip › Supplementary Materials Figures S1-S5 and Tables S1-S3.pdf]

Supplementary Materials

Targeted Proteomics Reveals Quantitative Differences in Low-Abundance Glycosyltransferases of Patients with Congenital Disorders of Glycosylation

Roman Sakson [1,2,*,†], Lars Beedgen [3,†], Patrick Bernhard [4], Keziban Merve Alp [5], Nicole Lübbehusen [1], Ralph Röth [6], Beate Niesler [6,7], Marcin Luzarowski [1], Olga Shevchuk [8], Matthias P. Mayer [1], Christian Thiel [3] and Thomas Ruppert [1,*]

[1] Zentrum für Molekulare Biologie der Universität Heidelberg (ZMBH), DKFZ-ZMBH Alliance, 69120 Heidelberg, Germany
[2] Heidelberg Biosciences International Graduate School (HBIGS), Heidelberg University, 69120 Heidelberg, Germany
[3] Center for Child and Adolescent Medicine, Department Pediatrics I, Heidelberg University, 69120 Heidelberg, Germany
[4] Institute for Surgical Pathology, Medical Center—University of Freiburg, Faculty of Medicine, University of Freiburg, 79106 Freiburg, Germany
[5] Max Delbrück Center for Molecular Medicine in the Helmholtz Association (MDC), 13125 Berlin, Germany
[6] nCounter Core Facility, Institute of Human Genetics, University Hospital Heidelberg, 69120 Heidelberg, Germany
[7] Interdisciplinary Center for Neurosciences, Heidelberg University, 69120 Heidelberg, Germany
[8] Department of Immunodynamics, Institute of Experimental Immunology and Imaging, University Hospital Essen, 45147 Essen, Germany
* Correspondence: r.sakson@hs-mannheim.de (R.S.); t.ruppert@zmbh.uni-heidelberg.de (T.R.)
† These authors contributed equally to this work.

Supplementary Materials and Methods: Page S-2

Supplementary References: Page S-4

Supplementary Figures S1–S5 Page S-5

Supplementary Tables S1–S3 Page S-10

Table S4 is provided as a separate Excel file.

Supplementary Materials and Methods

Cell Culture

All cell lines were maintained at 37 °C in a humidified atmosphere under 5 % CO_2. Fibroblasts were cultured in Dulbecco's modified Eagle's medium (high glucose; Life Technologies) supplemented with 10 % FCS (PAN Biotech), 100 U/mL penicillin, and 100 μg/mL streptomycin and harvested using a cell scraper. Unlabeled HEK 293T and HeLa cells were cultured in Dulbecco's modified Eagle's medium (Thermo Fisher Scientific) supplemented with 10 % FBS (Thermo Fisher Scientific), 100 U/mL penicillin, and 100 μg/mL streptomycin. All HEK 293T cells and HeLa cells were harvested using 0.25 % (v/v) trypsin-EDTA solution.

Membrane Protein Enrichment Procedure and Data Analysis

The preparation of the membrane protein fraction was performed as previously described [1]. "In short, cell pellets of isotopically labelled HEK 293T cells were resuspended in the hypotonic buffer (10 mM HEPES-KOH, 1.5 mM $MgCl_2$, 10 mM KOAc, 0.5 mM DTT, pH 7.4, 8 μM pepstatin, 20 μM leupeptin, 1 mM aprotinin, 1 mM phenylmethylsulfonyl fluoride). Cells were lysed by passing the suspension through a 27G syringe needle for five times (Figure S1). After removing cell debris and remaining intact cells by centrifugation at 1,000 g for 5 min at 4 °C, the post-nuclear supernatant was centrifuged at 100,000 g for 30 min at 4 °C through a low-salt sucrose cushion (500 mM sucrose, 50 mM HEPES-KOH, pH 7.4, 50 mM KOAc, 5 mM $Mg(OAc)_2$). The resulting pellet was resuspended in the rough microsomal buffer (250 mM sucrose, 50 mM HEPES-KOH, 50 mM KOAc, 2 mM $Mg(OAc)_2$, 1 mM DTT, pH 7.4) before increasing the salt concentration up to a final concentration of 500 mM KOAc and 50 mM EDTA in order to strip away remaining ribosomes of the rough ER. After incubating the sample for 20 min on ice, the ribosomes were removed by centrifugation through a high-salt sucrose cushion (500 mM sucrose, 50 mM HEPES-KOH, pH 7.4, 500 mM KOAc, 5 mM $Mg(OAc)_2$). The resulting membrane pellet thereof was resuspended in a 100 mM sodium carbonate solution to break microsomal membranes and remove the ER lumen before performing the last centrifugation step at 140,000 g for 30 min at 4 °C through an alkaline sucrose cushion (125 mM sucrose, 100 mM Na_2CO_3)." The resulting pellet was resuspended in RIPA buffer, aliquoted, snap-frozen in liquid nitrogen, and stored at - 80 °C.

Two independently enriched membrane fraction (MF) preparations were compared to a whole cell lysate (WCL, lysed with RIPA buffer), in technical LC-MRM measurement triplicates, respectively (n=6 for MF and n=3 for WCL). Cells were harvested and pooled from ten confluent 150-mm cell culture plates per enrichment. 4 μg of total protein from the WCL or the MF were used for LC-MRM analysis. The resulting peak areas were normalized to 8 iRT peptides as global standards and protein fold changes were calculated in Skyline 20.1.0.155 (Figure S2).

Peptide Candidate Selection Procedure

Peptides were selected from an *in silico* digest of the human canonical proteome retrieved from the UniProtKB database [2] (www.uniprot.org, Proteome ID: UP000005640, downloaded on 16.04.2018). The digest was performed in Skyline 64-bit version 20.1.0.155 accordingly to the following criteria: all peptides were tryptic, 7-21 amino acids in length, without missed cleavages and

methionine residues. Furthermore, chosen peptides were checked for uniqueness regarding the respective protein against the human canonical background proteome. Availability of existing mass spectrometric data for each respective peptide was checked by using our own previous data-dependent acquisition data as well as the publicly available repository SRMAtlas (www.srmatlas.org) [3]. Whenever more than ten candidates remained after filtering steps, the detectability of each peptide was checked by using the CONSeQuence web interface from the University of Manchester (http://king.smith.man.ac.uk/CONSeQuence) [4], where a score of 3-4 was considered as a detectable peptide. The merged information from the mentioned sources was used as basis for the selection of the most promising peptides to serve as proxies for the protein of interest.

MRM-Initiated Detection and Sequencing (MIDAS) Settings and Data Analysis

MIDAS was used to independently validate the correct amino acid sequence and to identify appropriate MRM transitions for the heavy labeled synthetic peptides (1 pmol per injection) in the presence of indexed Retention Time (iRT) peptides [5] as described [6]. iRT peptide concentrations were adjusted to produce MRM-signals of comparable intensities. LC-MIDAS measurements were performed on the QTRAP® 5500 (Sciex) system in the positive ion mode with a maximum of 50 transitions per sample injection. Briefly, we used 30 ms dwell times for unscheduled MRM, a mass tolerance of 0.25 Da and an intensity threshold of 3000 counts/s, followed by up to two Enhanced Product Ion scans in the range of 230 – 1000 Da. Dynamic exclusion was set to 9 s. 9 out of 10 iRT peptides were set to the permanent exclusion list, whereas the one remaining iRT peptide (DGLDAASYYAPVR) was used as positive control for the following database search. Resulting MS2 spectra were further preprocessed using the mascot.dll script within Analyst® 1.6.2 (Sciex) to generate an mgf-file. 2+ and 3+ were used as default precursor charge states, peaks with intensity of less than 1 % of the maximum intense peak were removed and spectra with less than 10 peaks were rejected. The generated mgf-file was then used to identify the respective precursors by performing a Mascot Database Search against the same canonical and isoforms containing human protein database that was used for peptide candidate selection, extended by common contaminants. Trypsin was set as digestion enzyme, with no allowed missed cleavages, a precursor (MS1) tolerance of 1 Da and a fragment (MS2) tolerance of 0.3 Da. Only doubly and triply positively charged precursor ions were allowed. Carbamidomethylation of cysteine was selected as a fixed modification. Methionine oxidation together with the selected labels for lysine $^{13}C_6$ $^{15}N_2$ (+ 8 Da) and arginine $^{13}C_6$ $^{15}N_4$ (+ 10 Da) were set as variable modifications. The results of the database search were exported as Mascot .dat files and inspected in Skyline 64-bit version 20.1.0.155 as spectral libraries together with the MRM extracted ion chromatograms. Precursors displaying very broad signals or showing identical signals at distinct retention times were excluded from further analysis.

Supplementary References

1. Avci, D.; Malchus, N.S.; Heidasch, R.; Lorenz, H.; Richter, K.; Nessling, M.; Lemberg, M.K. The intramembrane protease SPP impacts morphology of the endoplasmic reticulum by triggering degradation of morphogenic proteins. *J Biol Chem* **2019**, *294*, 2786-2800, doi:10.1074/jbc.RA118.005642.
2. Consortium, T.U. UniProt: the Universal Protein Knowledgebase in 2023. *Nucleic Acids Research* **2022**, *51*, D523-D531, doi:10.1093/nar/gkac1052.
3. Kusebauch, U.; Campbell, D.S.; Deutsch, E.W.; Chu, C.S.; Spicer, D.A.; Brusniak, M.Y.; Slagel, J.; Sun, Z.; Stevens, J.; Grimes, B.; et al. Human SRMAtlas: A Resource of Targeted Assays to Quantify the Complete Human Proteome. *Cell* **2016**, *166*, 766-778, doi:10.1016/j.cell.2016.06.041.
4. Eyers, C.E.; Lawless, C.; Wedge, D.C.; Lau, K.W.; Gaskell, S.J.; Hubbard, S.J. CONSeQuence: prediction of reference peptides for absolute quantitative proteomics using consensus machine learning approaches. *Mol Cell Proteomics* **2011**, *10*, M110 003384, doi:10.1074/mcp.M110.003384.
5. Escher, C.; Reiter, L.; MacLean, B.; Ossola, R.; Herzog, F.; Chilton, J.; MacCoss, M.J.; Rinner, O. Using iRT, a normalized retention time for more targeted measurement of peptides. *Proteomics* **2012**, *12*, 1111-1121, doi:10.1002/pmic.201100463.
6. Unwin, R.D.; Griffiths, J.R.; Whetton, A.D. A sensitive mass spectrometric method for hypothesis-driven detection of peptide post-translational modifications: multiple reaction monitoring-initiated detection and sequencing (MIDAS). *Nat Protoc* **2009**, *4*, 870-877, doi:10.1038/nprot.2009.57.

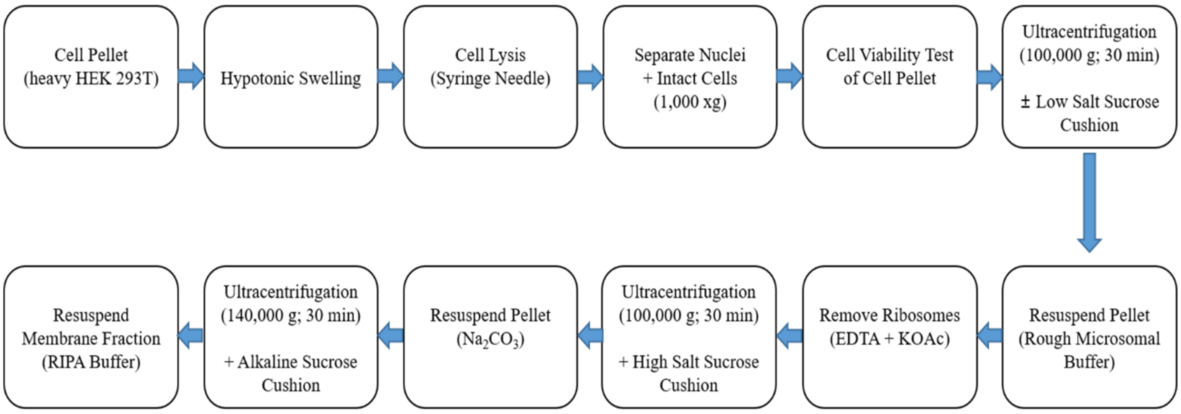

Figure S1. Preparation of a microsomal membrane fraction from HEK 293T cells.

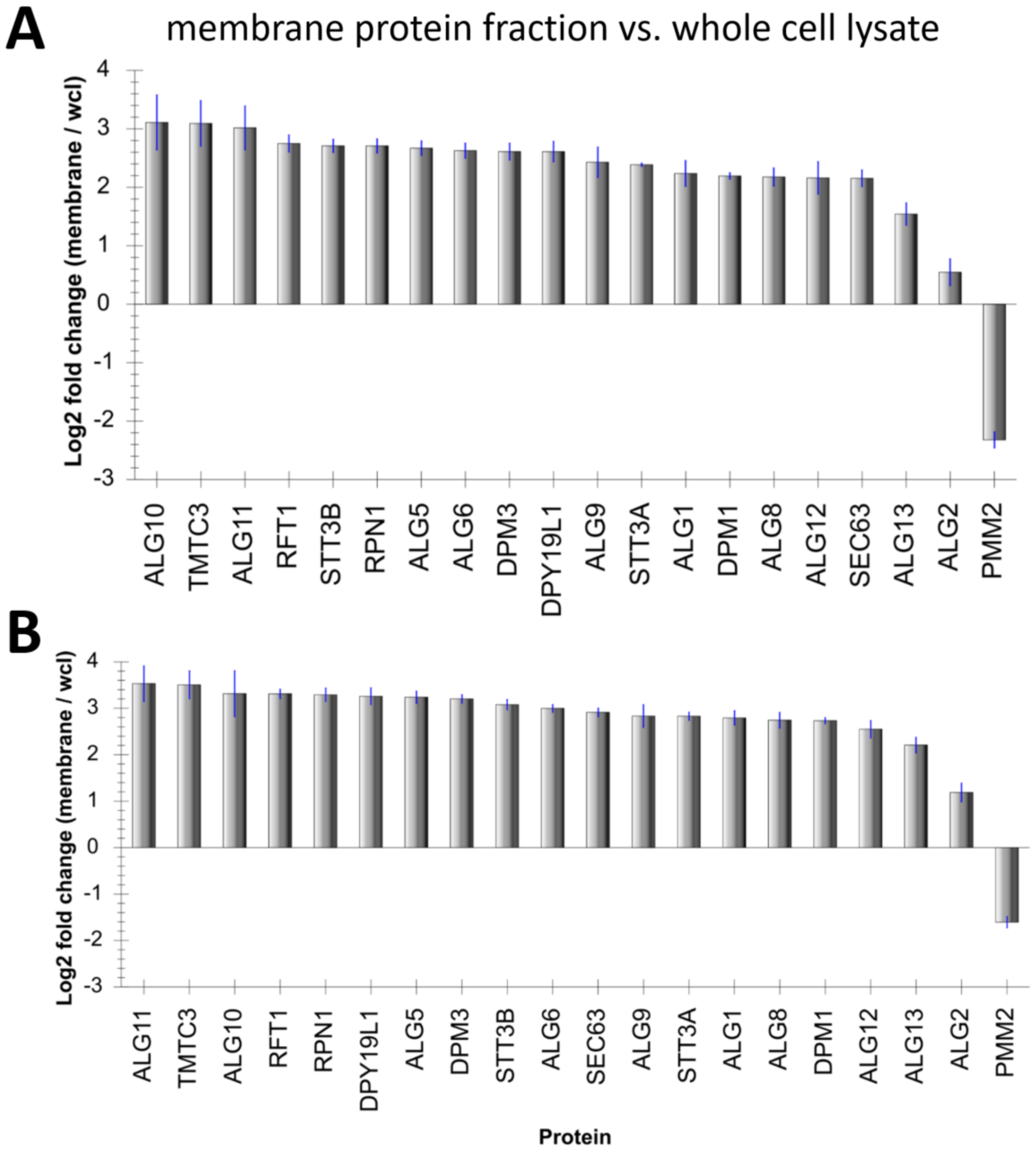

Figure S2. Results of two independent preparations of an enriched membrane protein fraction (**A-B**). Proteins are ordered with descending fold change from left to right. Both enrichment experiments show similar results: all monitored proteins except for the cytosolic PMM2 were significantly enriched in the membrane protein fraction (all adjusted p-values < 0.01). Error bars show 95 % confidence intervals (n=3 technical replicates per enrichment).

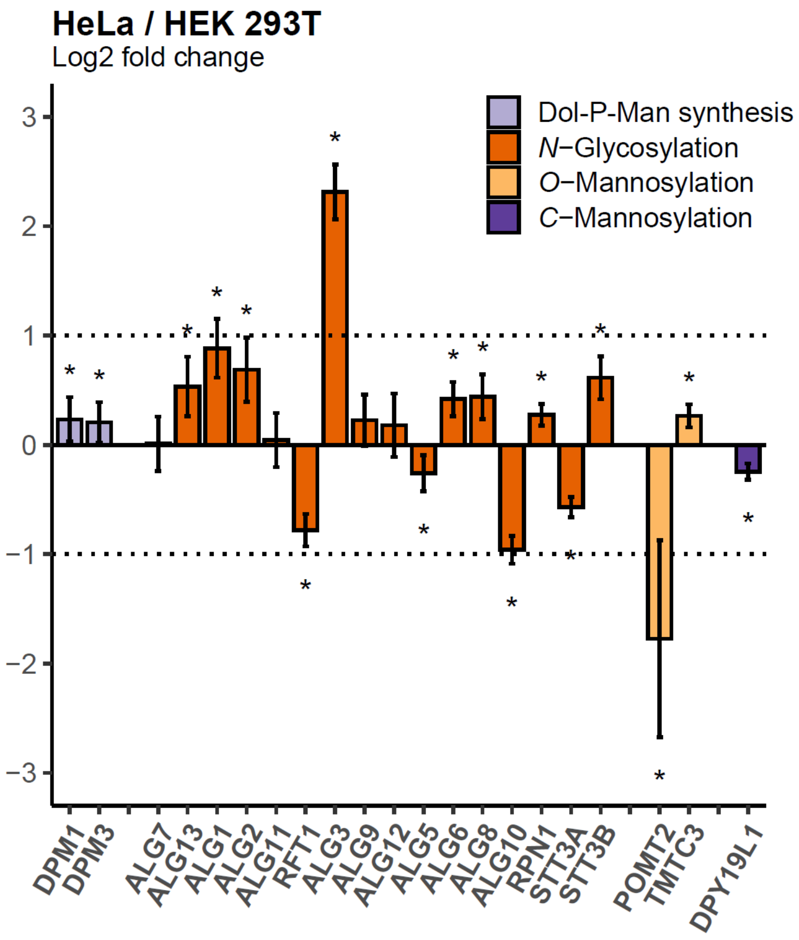

Figure S3. Relative quantification of 21 target proteins in HeLa cells (n=3) compared with HEK 293T cells (n=3). Ratios between whole cell lysates and the internal standard were obtained and used to calculate fold changes between HeLa cells and HEK 293T. Dotted lines indicate the range between +/- two-fold change in relative protein abundance. Error bars show 95 % confidence intervals and stars indicate adjusted p-values ≤ 0.05.

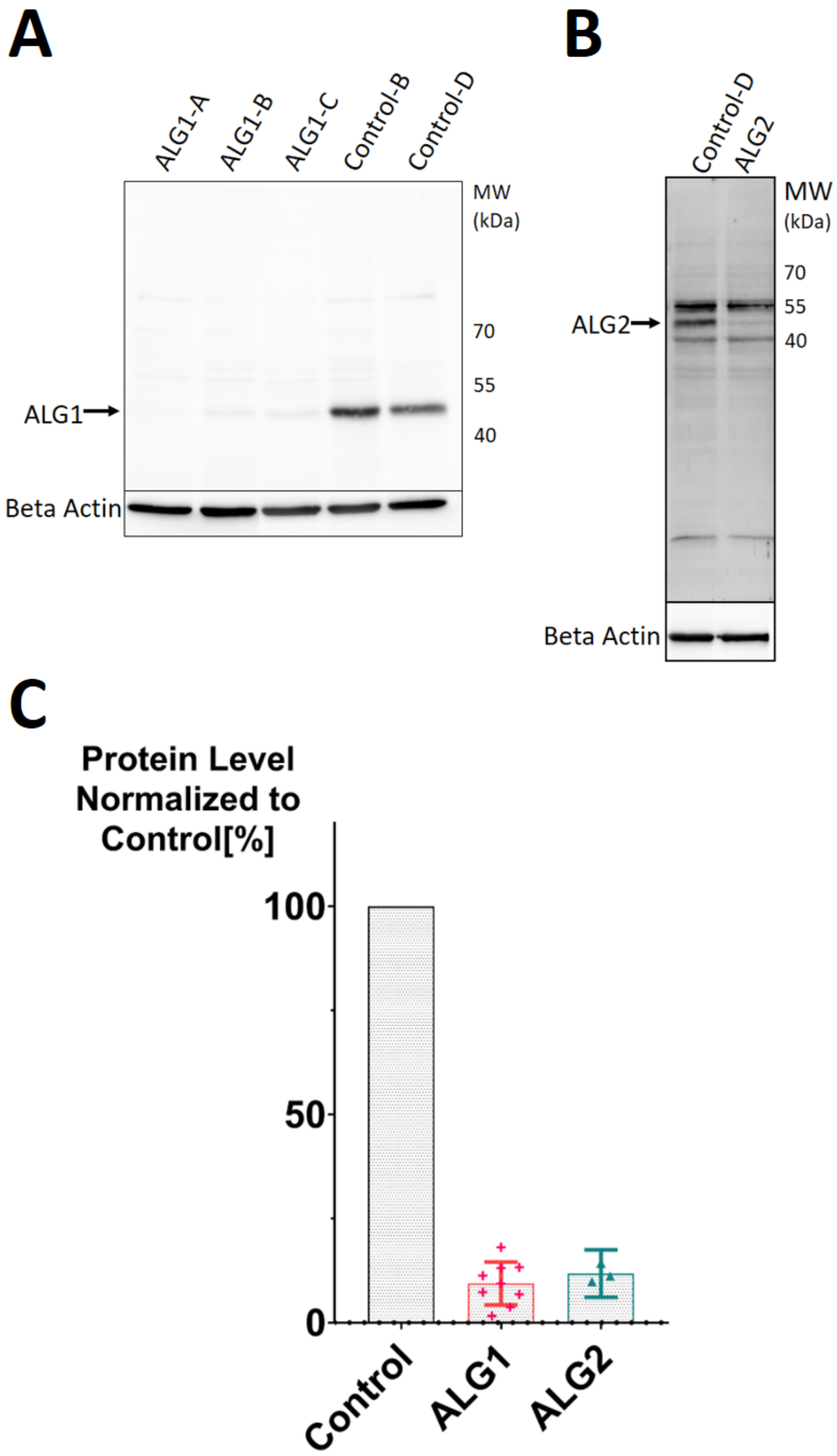

Figure S4. Validation of quantitative LC-MRM results by Western blot (WB) analysis. Representative ALG1 blots for three ALG1-CDG patients and two control fibroblast cell lines (**A**) as well as WB for ALG2 for one ALG2-CDG patient and one control cell line (**B**) are shown. (**C**) Quantitative result summary for all WBs performed (n=3). Error bars show 95 % confidence intervals.

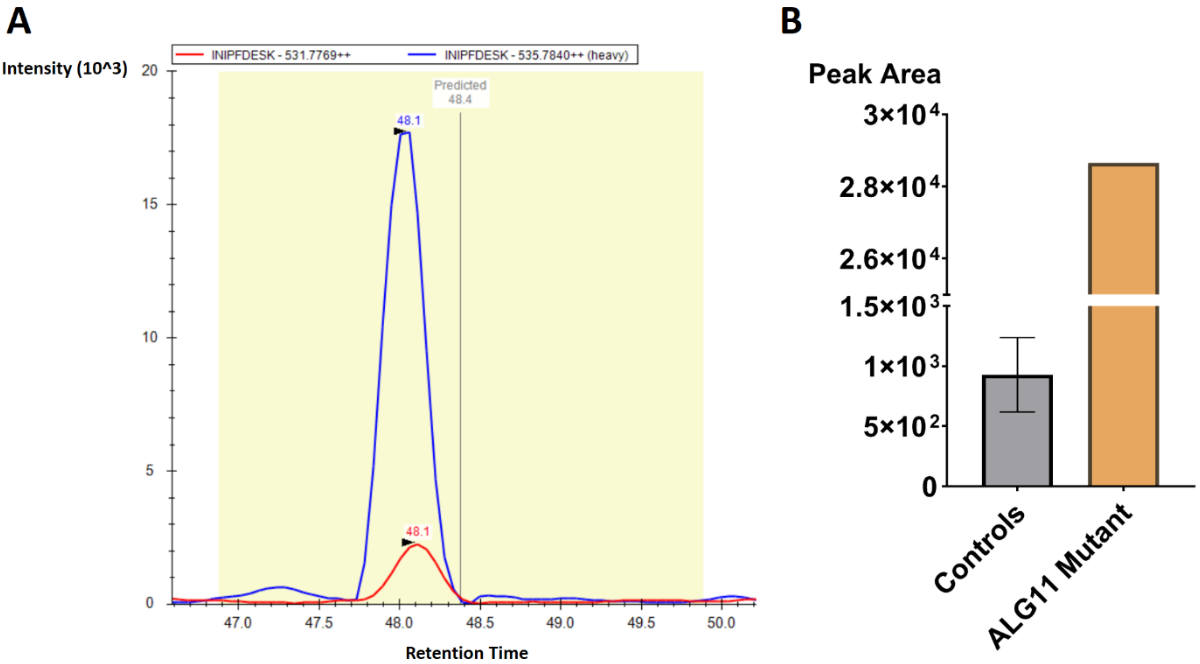

Figure S5. The peptide INIPFDE**S**K containing an amino acid substitution is specifically detected in the respective patient ALG11_I-CDG. (**A**) Elution profile of the synthetic, heavy labeled peptide INIPFDESK (blue) validates the signal (red) found in the patient fibroblast sample in Freiburg as the mutated target peptide. The retention time was also excellently predicted by the iRT calculated in Heidelberg. (**B**) The validated signal from figure part (**A**) is present in the patient and essentially absent in controls. Error bar shows standard error of mean.

Table S1. Overview of the 173 peptides detectable by the established MRM assay. All proteins and peptides are listed alphabetically. The canonical sequence from the UniProtKB is meant unless indicated otherwise.

Protein name (UniProtKB Accession Number)	Evaluated peptides
**ALG1** (Q9BT22)	AVTVYDKPASFFK; EDLADNWHIR; ETPLDLQHR; HVVAV-VLGDVGR; IQIVGLTELQSLAVGPR; SEPEDPVTER; VFQYGVK
**ALG10** (Q5BKT4 or Q5I7T1)	GPFAEFR; HYTFYVWK; TFLSLVWK; YFILPYVIYR
**ALG11** (Q2TAA5)	INIPFDELK; LIHPVQFVFLR; LVLIGGCR; NQNIGFNNAAFITR; VLWCALR
**ALG12** (Q9BV10)	FLQVNSAWR; GCSYLLNNYK; QLTWPEGK; VLWYNTVLNK
**ALG13** (Q9NP73-2)	ADLVISHAGAGSCLETLEK; FSAFLDK; GTVVPEPFSTESFTLDVYR; IESLGYNR; LILQIGR
**ALG14** (Q96F25)	INSFELDR; LLGSLSNAYSPR
**ALG2** (Q9H553)	CAGDWLPR; FLLLSINR; FSPEAFTEQLYR; GAAVCAYVR; ILFYCH-FPDLLLTK; IWTAHYDPGHCFAESR; VLENVEHYQELK
**ALG3** (Q92685)	FLPEALFLHR; TGESILSLLR; VAYTEIDWK; WLTHLLR
**ALG5** (Q9Y673)	ETLPSIWDSPTK; QLSVVVPSYNEEK; TFSSLHVER; YLTGAWR
**ALG6** (Q9Y672)	EQTLQVLR; FINPDWIALHTSR; HWQEITFNLPVK; SFSISVR; TSEEELQLK; YLPCFTFLSR
**ALG7** (Q9H3H5)	SLSFLGTFILK; VLGPIHER
**ALG8** (Q9BVK2)	CLLIPTYHSTDFEVHR; FLDPNNIPK; HIYLYVAPAYGVYLLR; VLSVIGLK
**ALG9** (Q9H6U8)	AGQVWAPEGSTAFK; GYHGPLDLYPEFYR; ILVFYFLR; SYAYLLLHAWPAAFHAR
**DPM1** (O60762)	DVAEQLEK; EGNFDIVSGTR; ENLPLIVWLLVK; FIPEFIR; GNGGVYGWDLK; LGGNEIVSFLK; QLNYTIGEVPISFVDR; YSVLLPTYNER
**DPM3** (Q9P2X0-2)	ELQSQIQEAR; FSFLLLR; VATFHDCEDAAR
**DPY19L1** (A0A1B0GW05)	AEPGAPPAGGGLGGR; DSKPHFTTVFQNSVYK; GASDVGAGEPGPER; HFSHLSTLER; IFGIADDAHIGNLLTSK; LSALRPIVNHPHYEDAGLR; LTEYPLVINTLK; TPLCNLLVK
**DPY19L3** (Q6ZPD9)	APEEVHALLR; ATEVSEDFPAQEENVK; AVYQIYAK; FGLGATR; NLPPYVAYFTR; TINLLQR; TLTNHPHYEDSSLR; VEFTIPLR

**DPY19L4** (Q7Z388)	ETVTLEDGR; INYSPYVNYFTR; ISDIPIPER; NENIYQIYSK
**PMM2** (O15305)	IEFYELDK; IGVVGGSDFEK; TIYFFGDK; VQEQLGNDVVEK; YDYVFPENGLVAYK
**POMT1** (Q9Y6A1-2)	DSWDILIR; ELHSPAQVDVSR; FVHVNTSAVLK; GSSHQQQVT-CYPFK; GYHGSTVWNVEEHR; IGAEYSSNVPVWSLR; LSGAHLPDWGYR; NVHDLPQDAWLR
**POMT2** (Q9UKY4)	DYNNLWIIK; ETLNSIWNVEDHINPK; HLYPEGIGAR; HNTNSDPLDPSFPVEFVR; LPAEVAGLSQVLLR; SGPGDGFFSSA-FQAR; TFFFDVHPPLGK
**RTF1** (Q96AA3)	ITDLLPNITR; ITQSLCFIHR; LQTLPVSR; LTWSFFK; NGAFINWK
**RPN1** (P04843)	ALTSEIALLQSR; ATSFLLALEPELEAR; DVPAYSQDTFK; FPLFGGWK; IILPEGAK; VTAEVVLAHLGGGSTSR
**SEC63** (Q9UGP8)	AYAALTDEESR; DDEAEWQELQQSIQR; ITHPVYSLYFPEEK; SPLLQLPHIEEDNLR; VLLLSHLAR; YSGDQILIR
**STT3A** (P46977)	EAYYWLR; ENDYYTPTGEFR; FGQVYTEAK; FHNWFDDR; FLAEE-GFYK; FYSLLDPSYAK; IIFDDFR
**STT3B** (Q8TCJ2)	EAYFWLR; ESDYFTPQGEFR; FESIIHEFDPWFNYR; FYS-LWDTGYAK; HLEEAFTSEHWLVR; NILDDFR; VTNIFPK
**TMTC1** (Q8IUR5)	AEILSPLGALYYNTGR; ALFNLGNLLK; HASALNNLGTLTR; LCTWLNR; SGVQTLPHNAK; SPQQPGSPQPSSLPGHPHR; VHYNYANFLK; VISELFFTK; WGIFTNDFWGK
**TMTC2** (Q8N394)	ALQLKPDDVITQSNLR; AWGNLGNVLK; FAEALHYYK; QILPTIYK; QNANGHSCLSDVEYQNSETK; SYRPLCTLSFR; TDHIPAHLTYGK; TLTFFYLPTK
**TMTC3** (Q6ZXV5)	ALPILEELLR; CLLETLALAPHEEYIQR; FDNPAAVSPTPTR; IAP-NHLNVYINLANLIR; ISSSSFIEPIFPTSK; LWNNVGHALENEK; SALFNLALLYSQTAK
**TMTC4** (Q5T4D3)	ANPNAASYHGNLAVLYHR; ESEALFLK; FEAAEQSYR; FNVLEIVQK; HVDALNAWR; HYEISLQLDPTASGTK; SALSVCPLNAK; SEEQLFR; WGHLDLAK

Table S2. Data analysis steps performed by the in-house developed R-script. All steps until step 8 (the adjustment of p-values for multiple hypothesis testing) are being performed for each protein target iteratively.

1) Load data regarding transition areas in, as well as information regarding assignment of replicate names from the Skyline export to conditions and the name of the reference protein to normalize to. Remove iRT peptides and not quantitative transitions. Truncated but quantitative transitions can be kept or removed, depending on the user's decision.
2) Transition areas that belong to the same protein within each replicate are grouped. Light and heavy areas are summed up separately.
3) Light and heavy protein areas are log-transformed.
4) In a first data normalization step to the internal heavy standard, light-to-heavy ratio for each protein within each replicate is calculated.
5) In a second data normalization step, all light to heavy protein ratios within a replicate are normalized by the respective light-to-heavy ratio of the reference protein (SEC63 in the provided R-script version). However, any other protein from the MRM assay can be defined by the user (this step can also be omitted entirely).
6) For each target protein, a pairwise group comparison between a treated condition and a control can be performed. Log-transformed, normalized protein ratios from replicates are grouped according to the two conditions for a linear regression: each protein replicate has its normalized area ratio as y-axis value. All control replicates have zero and all treatment replicates have one as the respective x-axis value.
7) A linear regression is performed, the slope of the obtained fit corresponds to the protein fold change (logarithmic scale). A p-value is also directly calculated during the regression procedure. Standard error of the slope and the number of replicates are used to calculate confidence intervals at a confidence level defined by the user (95 % as default).
8) Obtained p-values are adjusted depending on the number of protein targets in the MRM assay.
9) Results can be exported as a txt-file and automatically visualized as bar plots.

Table S3. List of used cell lines.

Cell Line	Origin	Source
HEK 293T	Human Embryonic Kidney	kindly provided by Dr. Marius Lemberg (ZMBH, Heidelberg)
HeLa CCL2™	Human Cervical Cancer	ATCC, Manassas, USA; kindly provided by Dr. Anne-Lore Schlaitz (ZMBH, Heidelberg)
PCS-201-010™ (Control-A)	Human Skin Fibroblasts, neonatal	ATCC, Manassas, USA
SCC058 (Control-B)	Human Skin Fibroblasts	Merck, Darmstadt, Germany
CCD-39Sk (Control-C)	Human Skin Fibroblasts	ATCC, Manassas, USA
N21 (Control-D)	Human Skin Fibroblasts	Internal control cell line University Hospital Heidelberg; kindly provided by PD Dr. Christian Thiel (University Hospital Heidelberg)
